# Evolutionary Strategies of Viruses, Bacteria and Archaea in Hydrothermal Vent Ecosystems Revealed through Metagenomics

**DOI:** 10.1371/journal.pone.0109696

**Published:** 2014-10-03

**Authors:** Rika E. Anderson, Mitchell L. Sogin, John A. Baross

**Affiliations:** 1 School of Oceanography and Astrobiology Program, University of Washington, Seattle, Washington, United States of America; 2 Josephine Bay Paul Center, Marine Biological Laboratory, Woods Hole, Massachusetts, United States of America; University of South Florida College of Medicine, United States of America

## Abstract

The deep-sea hydrothermal vent habitat hosts a diverse community of archaea and bacteria that withstand extreme fluctuations in environmental conditions. Abundant viruses in these systems, a high proportion of which are lysogenic, must also withstand these environmental extremes. Here, we explore the evolutionary strategies of both microorganisms and viruses in hydrothermal systems through comparative analysis of a cellular and viral metagenome, collected by size fractionation of high temperature fluids from a diffuse flow hydrothermal vent. We detected a high enrichment of mobile elements and proviruses in the cellular fraction relative to microorganisms in other environments. We observed a relatively high abundance of genes related to energy metabolism as well as cofactors and vitamins in the viral fraction compared to the cellular fraction, which suggest encoding of auxiliary metabolic genes on viral genomes. Moreover, the observation of stronger purifying selection in the viral versus cellular gene pool suggests viral strategies that promote prolonged host integration. Our results demonstrate that there is great potential for hydrothermal vent viruses to integrate into hosts, facilitate horizontal gene transfer, and express or transfer genes that manipulate the hosts’ functional capabilities.

## Introduction

The deep subsurface below hydrothermal systems hosts a high diversity of archaea, bacteria, and viruses that must tolerate extremely variable environmental conditions. High-temperature, reduced hydrothermal fluids mix with cold, oxidized seawater both above and below the seafloor to establish strong gradients in temperature, pH, and chemical and mineralogical composition [1]–[5]. Wide variations in environmental parameters can occur over centimeter scales. Constant fluid flux throughout and above the subsurface transports organisms from one region to the next, exposing them to a range of environmental conditions. Gradients that dominate this environment create a highly diverse microbial community consisting of both archaea and bacteria [6]. Physical and chemical parameters vary according to fluid mixing and volcanic activity, leading to niche partitioning in microbial communities across both space [1] and time [7], [8]. Moreover, hyperthermophiles are routinely cultured from fluids that exit at low temperatures (5–30°C) [9], [10], indicating that organisms in vent systems are frequently flushed from their native habitats, most likely from the deep subsurface. Microbial communities in hydrothermal systems are known to form biofilms that coat mineral surfaces, including within high-temperature chimney structures [5]. Such biofilms, which are likely to occur within the subsurface as well, host high-density communities with potentially high contact rates between organisms.

The dynamic, diverse and dense nature of this habitat should foster frequent exchange of genes within the microbial community. Previous work with vent samples has shown that the genes responsible for this process, including transposases and integrases, were observed to occur at high frequency in cellular metagenomes from hydrothermal systems as compared to other environments [11], [12]. Analysis of fully sequenced genomes of thermophiles, including many from vent systems, suggests that gene transfers occur more frequently among thermophiles than mesophiles or psychrophiles [13], [14] and that these transfers sometimes cross domains [13]–[15]. The prevalence of horizontal gene transfer in vent systems may expand the functional repertoire of a given species, expanding the pangenome and providing access to different ecological niches. This expanded metabolic flexibility would provide a strong advantage in hydrothermal vent environments where fluid flux and environmental gradients expose communities to wide extremes in temperature, pH, and chemical composition.

Here, we use comparative metagenomics to elucidate the role that viruses play in facilitating gene flow and manipulating host genetic potential in hydrothermal systems. Viruses are known to play pivotal roles in the transfer of genes and the alteration of host phenotype, particularly in the pelagic oceans (see Breitbart 2012 [16] for review). Bacterial and archaeal viruses introduce foreign genetic material through transduction and expression of virally encoded genes during infection. Transduction, or virally-mediated horizontal gene transfer, occurs on a massive scale in the surface oceans. Up to 10^14^ transduction events can occur per year in Tampa Bay estuary [17], and virus-like particles that serve as gene transfer agents (GTAs) may boost these transduction rates by one million-fold [18]. Viruses are known to encode auxiliary metabolic genes, or AMGs, which play critical roles in facilitating biochemical or metabolic processes [19]. For example, cyanophage transcribe and express photosynthesis genes during lytic infection of their cyanobacterial hosts [20]–[23], potentially to support the host during infection, or to redirect host metabolism to support phage deoxyribonucleotide biosynthesis [24]. Lysogenic viruses can have similar impacts on their hosts: the expression of genes encoded by integrated viruses (also known as proviruses, or prophage in bacteria) can manipulate host phenotype, such as in the case of the cholera toxin expressed by a prophage integrated in the *Vibrio cholerae* genome [25]. Selection should favor expression of genes within lysogenic viruses that enhance host fitness while the virus is integrated in the genome. For example, it has been hypothesized that proviruses express genes that suppress host metabolism to conserve resources under low-energy or low-nutrient conditions [26].

Despite increasing evidence that viruses play a crucial role in manipulating host genotype and phenotype in the surface oceans, this phenomenon has yet to be explored in the dynamic environment of hydrothermal vents. Viruses are abundant in hydrothermal systems [27] and have the potential to infect many different taxa of bacteria and archaea [3]. It has been suggested that up to 80% of archaea and bacteria in the deep ocean contain proviruses in their genomes [28]. Induction experiments have suggested that proviruses are particularly abundant in the genomes of archaea and bacteria from hydrothermal vent fluids compared to those in water from the deep ocean or the deep chlorophyll maximum [29]. Considering the abundance of viruses in these systems, and lysogenic viruses in particular, several questions arise: do these viruses transfer genes between hosts? Do they express fitness factors while integrated in the host genome? If so, which genes are expressed? Do viruses contribute to host genomic plasticity and facilitate their adaptation to changing conditions? Does selection act differently on viral genes compared to cellular genes?

To address these questions, we used a cultivation-independent approach that provides a community-wide perspective of both the viral gene pool and the bacterial and archaeal gene pool (hereafter referred to as the “cellular” gene pool) in hydrothermal systems. Specifically, we analyzed the unamplified viral and cellular metagenomes of high-temperature diffuse flow hydrothermal fluid from Hulk hydrothermal vent in the Main Endeavour Field on the Juan de Fuca Ridge. We compared the relative content of each of these gene pools and inferred the modes of genetic interaction between viruses and their hosts. This analysis focused on a unique fluid sample that contained organisms native to a wide range of ecological niches within the gradient-dominated hydrothermal environment, all with the potential to come into contact through constant fluid flux. Given the potential for gene and viral exchange across these niches, these metagenomes can provide insights into interactions within the diverse communal gene pool of the hydrothermal vent microbial community.

Comparative analysis of the cellular and viral metagenomes from this sample addressed whether viruses have the potential to manipulate the physiology or metabolism of their hosts. The presence of genes facilitating horizontal gene transfer and lysogenic virus integration described the genetic potential for these processes in the vent environment. We compared the relative abundance of genes in the viral and cellular gene pools in order to determine the types of genes enriched in the viral gene pool relative to the cellular gene pool. Finally, we asked how evolution has shaped the viral and cellular gene pools by examining relative selection pressures on viral and cellular genes. Together, these analyses provide insight into the broader question of how evolution has shaped the genomes of viruses and their hosts in some of the more extreme environments of the planet.

## Materials and Methods

### Sample collection and DNA extraction

We collected a 170-L hydrothermal vent fluid sample from Hulk vent at the Main Endeavour Field on the Juan de Fuca Ridge (47°57.00′ N, 129°5.81′ W) as described previously [3]. No specific permissions were required for these locations or sampling activities. The vent fluid was obtained using a large barrel sampler equipped with two 100-L sterile bags. We placed the sample collection funnel atop a region of diffuse venting, adjacent to a colony of tube worms on the side of a large sulfide structure. While the tube worms were surrounded by fluid at measured temperatures of 13–30°C, the average temperature of the metagenome fluid sample was calculated from its silica chemistry to be about 125°C [3]. This result indicates that we most likely sampled fluid ranging from cool background seawater to high-temperature hydrothermal fluid (up to 300°C) from the sulfide structure adjacent to the sample site, illustrating the dynamic fluid flux of these systems. The organisms collected in the sample therefore represent a range of habitats in the hydrothermal environment, including psychrophiles, mesophiles and thermophiles, and aerobic, microaerophilic, and anaerobic organisms. Some of these organisms may have been derived from deep subsurface fluids, whereas others from entrained seawater. Having been sampled from the same site, these organisms have the potential to come into contact within the vent environment due to dynamic fluid flux. We included available metadata about this vent sample in Table S1.

We collected the cellular fraction by filtering the 170 L of hydrothermal vent fluid through three 0.22 µl Steripaks (Millipore, USA) while the sample and filtrate were held on ice. The filtrate was retained for subsequent virus sampling. Filters were frozen at –80°C while shipboard and until sample processing. We extracted DNA from one Steripak using a modified DNA extraction procedure described by Anderson *et al.* [1]. Briefly, DNA extraction buffer (0.1 M Tris-HCl, 0.2 M Na-EDTA, 0.1 M NaH2PO4, 1.5 M NaCl, and 1% cetyltrimethylammonium bromide) was added to each filter, then the filters were capped and freeze-thawed five times. Lysozyme (50 mg/mL solution), proteinase K (1% solution), and SDS (20% solution) were added to each filter and incubated. Lysate was removed from filters and centrifuged; DNA was extracted from the supernatant using a phenol/chloroform/isoamyl extraction method described by Anderson *et al.* [1].

For virus collection, we concentrated the sample filtrate using tangential flow filtration (30 kDa cutoff) to approximately 400 mL in a 4°C cold room, and froze concentrated filtrate into six aliquots at –80°C until further processing. One aliquot was further concentrated by adding 10% w/v polyethylene glycol 8000 (PEG), incubating overnight at 4°C, and centrifuging at 13 000×g for 50 min. The pellet was resuspended in Tris-EDTA buffer and incubated for 15 min with 0.7 volume of chloroform to lyse any remaining cellular contamination. Free DNA was removed by incubating with 10% DNAse I for 2 h at 37°C, then inactivated by adding EDTA to a final concentration of 0.02 M. The QIAamp MinElute Virus Spin Kit (Qiagen) was used to extract the viral DNA, yielding approximately 90 ng, which was not amplified for downstream sequencing. PCR tests of extracted viral DNA using universal 16S archaeal and bacterial primers showed no amplification from contaminating cellular 16S rRNA, whereas positive controls of DNA extracted from *E. coli* showed successful amplification.

### Metagenomic sequencing

The viral metagenome was generated on a Roche Genome Sequencer FLX (GSFLX) with GS FLX Titanium 454 sequencing protocols by the Broad Institute. For the cellular metagenome, libraries were created using the Nexterra transposon-mediated method (Epicentre) at the Josephine Bay Paul Center at the Marine Biological Laboratory, then sequenced using Roche Titanium 454 sequencing protocols on a GSFLX. Both metagenomes are publicly available on the MG-RAST v3 database [30], with accession numbers 4469452.3 for the viral metagenome and 4481541.3 for the cellular metagenome. The viral metagenome was previously described in Anderson et al. [3] and was uploaded under MG-RAST v2, and is still available at 4448187.3.

We used TagCleaner [31] to trim tags from the 5′ end of each sequence in the cellular metagenome. Assembly of both the viral and cellular metagenomes was conducted in Geneious [32] using the “Medium Sensitivity” method, with a word length of 14, a maximum gap size of 2, maximum gaps per read of 15, and maximum mismatches of 2. To classify sequences using di, tri, and tetranucleotide analysis, we created a boutique database of bacterial and archaeal virus sequences (Table S2) as a training set to accompany the existing cellular dataset in PhylopythiaS [33], which was used to identify archaea, bacteria, archaeal viruses and bacterial viruses. Metagenomes were assembled in Geneious prior to classification with PhylopythiaS; only contigs over 1000bp in length were used.

### Enrichment of proviruses and mobile genetic elements

To identify the numbers of reads in each metagenome matching lysogenic viruses, metagenomes were compared to a database of sequences from the “Prophages” category in the ACLAME database [34]. To assess abundance of mobile genetic elements, we compared all metagenomes to a dataset of Pfam seed sequences [35] matching transposases, recombinases, resolvases and integrases, listed in Table S3, using tblastn with an e-value cutoff of 10–^5^. The number of unique reads with a match to a sequence from the query sequence collection was tallied and normalized to the number of reads in the metagenome. Only metagenomes generated with 454 pyrosequencing were used for the analysis, so that all metagenomic reads had a length ranging from approximately 100 to 300 bp.

### Relative enrichment of gene categories

We tallied gene categories by adding “abundance” counts for each functional category as defined by the KEGG Orthology database [36], the Clusters of Orthologous Groups (COG) database [37], or the SEED Subsystems database [38] in MG-RAST v3, using an e-value cutoff of 10^−3^. For the combined analysis of 20 cellular metagenomes and 23 viral metagenomes, all abundance counts for either viral or cellular metagenomes were tallied together. We used Xipe-Totec [39], a nonparametric method of statistical analysis using a difference of medians analysis, to determine whether abundance differences were statistically significant. For this analysis, we used a confidence level of 95% and a sample size of 5000 to determine significance.

### Fragment recruitment

Cellular metagenomic reads were recruited to genomes of hydrothermal vent isolates using NUCmer, part of the MUMmer 3.0 package [40], with the following parameters for the command line: -minmatch 10 -breaklen 1200 -maxgap 1000 -mincluster 50. Fragment recruitments were visualized using mummerplot in the MUMmer package. Coverage plots were created by using the show-coords command in MUMmer, then in-house Python scripts were used to calculate coverage for each base pair position. Coverage plots were created with a convolution function in numpy, using a moving average window size of 50000.

### Calculation of dN/dS

Prior to calculation of dN/dS ratios for genes mapped by each metagenome, metagenomes were subjected to stringent error filtering using Prinseq [41] with the following parameters: minimum sequence length of 60bp; minimum mean quality score of 30; maximum number of allowed Ns per sequence of 4; and low-complexity threshold of 70 (using Entropy). The dN/dS ratio measures selection pressures by calculating whether the number of non-synonymous substitutions (dN) in a gene is greater or fewer than the number expected by chance compared to the number of synonymous substitutions (dS). A majority-rule consensus was calculated from the mapped reads; the number of possible synonymous or nonsynonymous substitutions was then tallied and compared to the number of actual synonymous and nonsynonymous substitutions.

We mapped reads from both the viral and cellular metagenomes to the vent isolate genomes using CLC Genomics Workbench with the criteria of 80% identity and 80% coverage, using previously established benchmarks [42]. Mapping results were exported in ACE format; dN/dS was calculated for each gene using the Python scripts described in Tai *et al.* [42]. Polymorphisms were only tallied for positions with a mapping depth of at least 5X; only genes with at least 100 nucleotides at 5X depth were included in the analysis. Redundant genes were deleted from the analysis; only the dN/dS value for the gene with higher coverage was retained. The files used to define gene coordinates were downloaded from JGI IMG, with the exception of *T. kodakarensis*, which was derived from a.gff file obtained from NCBI. The 95% confidence interval was calculated for all genes mapped by the viral and cellular metagenomes with dN/dS less than 1 (subject to purifying selection) using alpha = 0.05.

## Results and Discussion

### General features of the metagenomes

**Figure 1:**
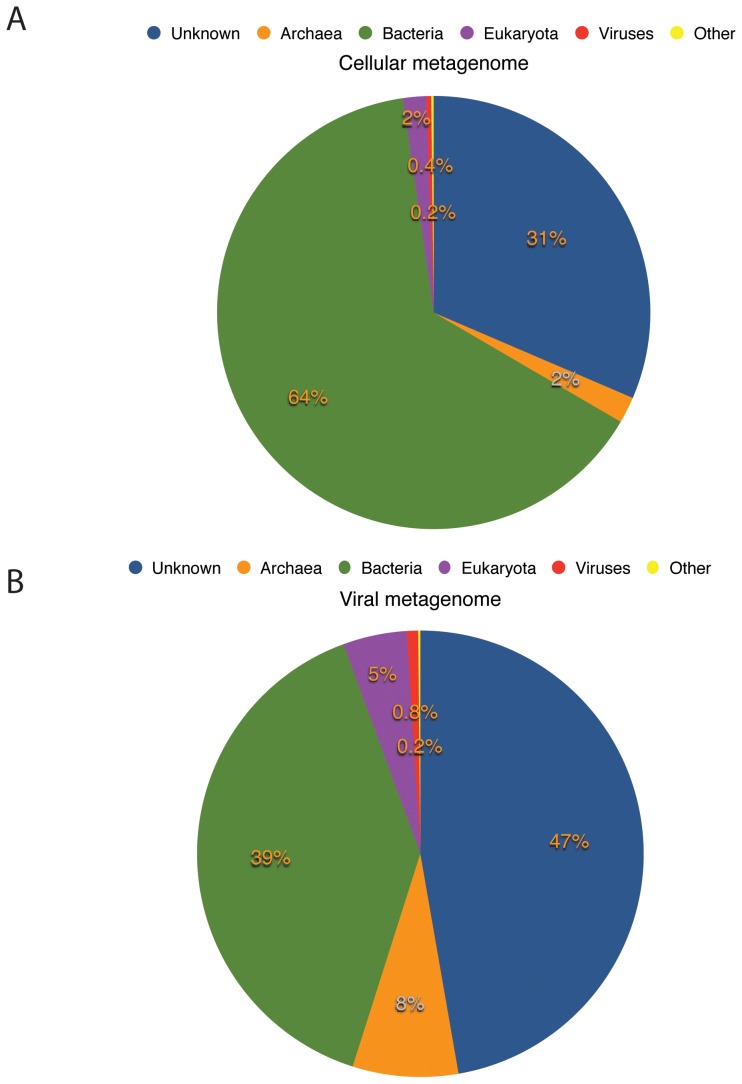
Pie charts showing breakdown of read classification for the cellular metagenome (A) and the viral metagenome (B) according to annotation by the M5NR database. Reads were annotated with a minimum e-value cutoff of 1e-05.

Metagenomic sequencing of the cellular and viral fractions from the large sample of hydrothermal fluid yielded a total of 808,051 and 231,246 sequence reads, respectively. The cellular metagenome contained reads from a wide variety of bacterial and archaeal taxa, including *Thermococcales*, methanogens, Marine Groups I and II, *Proteobacteria, Bacteroidetes, Firmicutes*, and many others, reflecting the wide range of ecological niches represented in the sample. Figure 1 indicates that approximately 31% of the cellular metagenome and 47% of the viral metagenome (virome) sequences had no matches to the M5NR database (classified here as “Unknown”) [43]. An analysis of the viral metagenome alone, including the taxa and diversity of viruses and their general host range, has been published previously [3]. Close matches between CRISPR spacers identified in the cellular metagenome and sequences in the viral metagenome suggest an active and relatively recent relationship between the two gene pools (see File S1). Classification of the cellular and viral metagenomes showed that only 2% of the reads from the cellular fraction matched archaeal genes, whereas nearly 8% of the viral metagenome reads matched archaeal genes (Figure 1). Nucleotide signature matching of assembled contigs longer than 1000 bp using PhylopythiaS [33] indicated that a disproportionate percentage of contigs in both metagenomes matched nucleotide compositional patterns of archaeal viruses, given that bacterial reads dominate both metagenomes (Figure S1). The percentage of contigs matching bacterial nucleotide compositional patterns was greater than the percentage of contigs with archaeal patterns by a ratio of 2.8 to 1 in the cellular metagenome. However, contigs matching bacterial virus patterns outnumbered contigs with archaeal virus patterns by only 1.8 to 1 in the viral metagenome. Taken together, these observations suggest that archaeal viruses may be disproportionally abundant in the vent habitat compared to the relative abundance of bacteria to archaea.

Assembly of viral metagenome contigs yielded many short, high-coverage (>20X coverage) contigs shorter than 6 kb (Figure S2). The cellular metagenome did not exhibit contigs with such high coverage and short length. In contrast, several contigs in the viral metagenome had relatively low coverage but were quite long. We annotated reads based on the best matches to the SEED database, and determined the taxonomy of each contig based on the annotation of the majority of the contained reads [3]. Many of the long, low-coverage contigs were annotated as bacterial or archaeal, whereas many shorter, high-coverage contigs were annotated as unknown (Figure S2).

We considered the long, low-coverage contigs with cellular annotation to be more likely to be derived from cellular contamination, whereas the high-coverage, shorter contigs with unknown annotation are more likely to be derived from viruses. These high-coverage contigs may include certain viral genomes that occurred at high frequency.

While we attempted to eliminate cellular contamination through size fractionation and chloroform and DNAse treatment prior to sequencing, and 16S PCR tests of extracted viral DNA returned no amplification of cellular material, we sought to further reduce cellular contamination *in silico.* We created a “viral subset” of the viral metagenome consisting of reads assembled into contigs with a coverage of 8 or greater, or assembled into contigs annotated as viral or unknown. The aim of producing a subset was to remove low-coverage contigs more likely to be derived from cellular contamination. We chose a coverage cutoff of 8 because most long contigs annotated as bacterial or archaeal had a coverage of approximately 8 or lower (Figure S2). The goal of this subset was not to generate a “pure” viral metagenome but rather to reduce the number of reads that may represent cellular contamination, which is frequently an issue in viral metagenomics [44], [45]. While the high percentage of unknown contigs in viral metagenomes makes any attempts at removing contamination difficult, this subset eliminates reads from low-coverage contigs with bacterial or archaeal annotation that are more likely to be derived from cellular contamination. Except in specific cases noted below, the analyses presented here or in the supplementary figures include both the original virome as well as the “viral subset” so that both may be compared.

### Assessing the potential for horizontal gene transfer

To assess the degree to which cells and viruses in hydrothermal ecosystems are capable of horizontal gene transfer or integration of proviruses, we determined the relative abundances of provirus genes (Table 1) and genes related to DNA transfer or mobilization (Table 2) in the hydrothermal vent cellular and viral metagenomes. We used the “Prophage” dataset in the ACLAME database [34] to identify provirus-related proteins in 22 pyrosequenced cellular metagenomes. These metagenomes were chosen to represent a range of aquatic and terrestrial environments, while controlling for sequencing method. Table 1 indicates that relative to the 22 cellular metagenomes, the hydrothermal vent cellular metagenome contained a high percentage of reads (approximately 4%) that match provirus-coding regions. This result provides compelling molecular evidence of abundant proviruses in cellular genomes in vents and complements descriptions of high proportions of lysogenic cells in vents and the deep ocean based upon mitomycin C induction experiments [28], [29]. We also analyzed the relative abundance of mobile genetic elements in 40 viral and cellular metagenomes, also selected to represent a range of environments, and controlled for sequencing method. We found a relative enrichment of mobile genetic elements in both the viral and cellular gene pools at Hulk hydrothermal vent (Table 2). Thus, not only do the genomes of archaea and bacteria in vents encode a higher abundance of mobile elements, on average, than genomes native to other habitats, but vent viruses encode a high abundance of mobile elements as well. These data suggest that selection has favored enrichment of mobile elements in these cellular and viral genomes, potentially leading to increased rates of horizontal gene flow among cells and viruses.

**Table 1:** Percent of reads in cellular metagenomes matching a protein in the “Prophage” grouping of the ACLAME database.

Metagenome	Reads	ACLAME Prophage hits	Percent reads	Biome	Sampling details	Reference	Accession number
Monterey Bay	192162	9759	5.08	Open ocean	Monterey Bay, California, surface waters, October	–	4443713.3
**Hulk hydrothermal vent**	**808051**	**32595**	**4.03**	**Hydro-thermal vent**	**Juan de Fuca Ridge, Northeast Pacific Ocean, 2198** **m depth**	**This study**	**4481541.3**
Glacial ice	1076539	40695	3.78	Glacial ice	Glacial ice of the Northern Schneeferner, Germany	Simon *et al.*, 2009	CAM_PROJ_IceMetagenome
North Atlantic Spring Bloom	257471	6913	2.68	Open ocean	Bermuda Atlantic Time-Series site	–	4443725.3
Human oral microbiota	339503	5596	1.65	Human	Dental plaque from 25 human volunteers	Belda-Ferre *et al.*, 2012	4447970.3
Healthy fish microbiota	51498	610	1.18	Fish	Healthy aquacultured fish, San Diego, CA	Angly *et al.*, 2009	4440055.3
Guaymas Basin	4970673	58638	1.18	Hydro-thermal vent	Hydrothermal plumes from Guaymas Basin, CA	Baker *et al.*, 2012	CAM_P_0000545
Cow rumen	320471	3678	1.15	Cow	Fiber-adherent microbiome from cow rumen	Brulc *et al.*, 2009	4441681.3
Healthy fish microbiota	60580	541	0.89	Fish	Samples from aquacultured fish gut contents	Angly *et al.*, 2009	4440059.3
Medium salinity saltern	108725	742	0.68	Salt water	Salinity 12–14% from solar salterns, California	Rodriguez-Brito *et al.*, 2010	4440425.3
Salton Sea	161912	992	0.61	Sediments	Sulfidic, anoxic sediments of the Salton Sea	Swan *et al.*, 2010	4440329.3
Tilapia fish pond	344260	1712	0.50	Fresh water	Water samples from aquaculture facility raising striped bass	Rodriguez-Brito *et al.*, 2010	4440440.3
Peru Margin 1mbsf	100093	489	0.489	Deep biosphere- marine sediment	Peru Margin ODP Leg 201 Site 1229, 1 meter below seafloor	Biddle *et al.*, 2008	4440961.3
Peru Margin 50mbsf	63258	288	0.455	Deep biosphere- marine sediment	Peru Margin ODP Leg 201 Site 1229, 50 meters below seafloor	Biddle *et al.*, 2008	4459941.3
Peru Margin 32mbsf	135429	479	0.354	Deep biosphere- marine sediment	Peru Margin ODP Leg 201 Site 1229, 32 meters below seafloor	Biddle *et al.*, 2008	4459940.3
Low salinity saltern	31948	111	0.35	Salt water	Salinity 6–8% from solar salterns, California	Rodriguez-Brito *et al.*, 2010	4440426.3
Microbialites	257573	802	0.311	Micro-bialites	Highborne Cay, Bahamas	Desnues *et al.* 2008, Dinsdale *et al.*, 2008	4440061.3
High salinity saltern	33356	98	0.29	Salt water	Salinity 27–30% from solar salterns, California	Rodriguez-Brito *et al.*, 2010	4440419.3
Peru Margin 16mbsf	121414	191	0.157	Deep biosphere- marine sediment	Peru Margin ODP Leg 201 Site 1229, 16 meters below seafloor	Biddle *et al.*, 2008	4440973.3
*Porites compressa* coral	1053275	947	0.0900	Coral	Samples collected at the Hawaii Institute for Marine Biology	Vega Thurber *et al.*, 2009	CAM_PROJ_CoralMetagenome
Soudan Mine	248038	193	0.0778	Deep biosphere- terrestrial mine	Water and sediments in mine, 714 m below surface, Soudan Mine, MN	Edwards *et al.*, 2006	4440282.3
Line Islands	178628	120	0.0672	Seawater	Water sampled near coral reefs, Christmas Island	Dinsdale *et al.*, 2008	4440041.3

Matches were found using tblastn with a minimum e-value of 10^−5^. All metagenomes listed here were generated with shotgun pyrosequencing.

**Table 2:** Percent of reads in cellular and viral metagenomes matching a mobile element.

Meta-genomes	Cellular or viral	Reads	Mobile elements	% reads	Biome	Sampling details	Ref	Accession number
Glacial ice	cellular	1076539	5598	0.52	Glacial ice	Glacial ice of the Northern Schneeferner, Germany	Simon *et al.*, 2009	CAM_PROJ_IceMetagenome
**Hydro-thermal vent**	**viral subset**	**64599**	**252**	**0.39**	**Hydro-thermal vent**	**Juan de Fuca Ridge, Northeast Pacific Ocean, 2198m depth**	**This study**	**4469452.3**
Healthy fish microbiota	cellular	51498	193	0.37	Fish	Healthy aquacultured fish, San Diego, CA	Dinsdale *et al.*, 2008	4440055.3
Healthy fish microbiota	cellular	60580	191	0.32	Fish	Samples from aquacultured fish gut contents	Angly *et al.*, 2009	4440059.3
**Hydro-thermal vent**	**cellular**	**808051**	**2539**	**0.31**	**Hydro-thermal vent**	**Juan de Fuca Ridge, Northeast Pacific Ocean, 2198m depth**	**This study**	**4481541.3**
Cow rumen	cellular	320471	976	0.30	Cow	Fiber-adherent microbiome from cow rumen	Brulc *et al.*, 2009	4441681.3
**Hydro-thermal vent**	**viral**	**231246**	**579**	**0.25**	**Hydro-thermal vent**	**Juan de Fuca Ridge, Northeast Pacific Ocean, 2198m depth**	**Anderson et al., 2011a**	**4469452.3**
Antarctic Lake summer	viral	30515	66	0.22	Fresh water	Freshwater oligotrophic lake, Byers Peninsula, Antarctica (summer)	Lopez-Bueno *et al.*, 2009	4441558.3
Reclaimed water	viral	1531954	2330	0.15	Fresh water	Viral fraction of reclaimed water	Rosario *et al.*, 2009	CAM_PROJ_ReclaimedWaterVirues
Monterey Bay	cellular	192162	278	0.14	Seawater	Monterey Bay, California, October 2000, surface waters	-	4443713.3
Human oral microbiota	cellular	339503	450	0.13	Human	Dental plaque from 25 human volunteers	Belda-Ferre *et al.*, 2012	4447970.3
Healthy fish microbiota	viral	55690	66	0.12	Fish	Samples from aquacultured fish gut contents	Angly *et al.*, 2009	4440065.3
Tilapia Pond	cellular	344260	352	0.10	Fresh water	Water samples from aquaculture facility raising striped bass	Rodriguez-Brito *et al.*, 2010	4440440.3
High salinity saltern	cellular	33356	33	0.10	Salt water	Salinity 27–30% from solar salterns, California	Rodriguez-Brito *et al.*, 2010	4440419.3
Guaymas Basin	cellular	4970673	4728	0.10	Hydro-thermal vent	Hydrothermal plumes from Guaymas Basin, CA	Baker *et al.*, 2012	CAM_P_0000545
Arctic Ocean	viral	688590	605	0.09	Sea-water	10–3246m, Fall 2002, Arctic Ocean	Angly *et al.*, 2006	4441621.3
Medium salinity saltern	cellular	108725	95	0.09	Salt water	Salinity 12–14% from solar salterns, California	Rodriguez-Brito *et al.*, 2010	4440425.3
Bay of British Columbia	viral	138347	107	0.08	Sea-water	0–245m, sampled over several dates, Bay of British Columbia	Angly *et al.*, 2006	4441623.3
North Atlantic Spring Bloom	cellular	257471	193	0.07	Seawater	Bermuda Atlantic Time-Series site	–	4443725.3
Peru Margin 1mbsf	cellular	100093	69	0.07	Deep biosphere- marine sediment	Peru Margin ODP Leg 201 Site 1229, 1 meter below seafloor	Biddle *et al.*, 2008	4440961.3
Salton Sea	cellular	161912	98	0.06	Sediments	Sulfidic, anoxic sediments of the Salton Sea	Swan *et al.*, 2010	4440329.3
Gulf of Mexico	viral	263908	153	0.06	Seawater	0–164m, sampled over several dates, Gulf of Mexico	Angly *et al.*, 2006	4441625.3
Micro-bialites	viral	621110	359	0.06	Micro-bialites	Pozas Azules, Mexico; Rio Mesquites, Mexico; Highborne Cay, Bahamas	Desnues *et al.*, 2008	4440320.34440321.34440323.3
Peru Margin 50mbsf	cellular	63258	28	0.04	Deep biosphere- marine sediment	Peru Margin ODP Leg 201 Site 1229, 50 meters below seafloor	Biddle *et al.*, 2008	4459941.3
Soudan Mine	cellular	248038	105	0.04	Deep biosphere- terrestrial mine	Water and sediments in mine, 714 m below surface, Soudan Mine, MN	Edwards *et al.*, 2006	4440282.3
Antarctic Lake spring	viral	31691	13	0.04	Fresh water	Freshwater oligotrophic lake, Byers Peninsula, Antarctica (spring)	Lopez-Bueno *et al.*, 2009	4441778.3
Coral	viral	36354	14	0.04	Coral	*Porites compressa* coral samples collected at the Hawaii Institute for Marine Biology	Vega Thurber *et al.*, 2009	4440374.3
Low salinity saltern	viral	56810	21	0.04	Salt water	Salinity 6–8% from solar salterns, California	Rodriguez-Brito *et al.*, 2010	4440420.3
Peru Margin 32mbsf	cellular	135429	49	0.04	Deep biosphere- marine sediment	Peru Margin ODP Leg 201 Site 1229, 32 meters below seafloor	Biddle *et al.*, 2008	4459940.3
Salton Sea	viral	27689	7	0.03	Sediments	Sulfidic, anoxic sediments of the Salton Sea	Swan *et al.*, 2010	4440328.3
Tilapia Pond	viral	231521	48	0.02	Fresh water	Water samples from aquaculture facility raising striped bass	Rodriguez-Brito *et al.*, 2010	4440439.3
Low salinity saltern	cellular	31948	6	0.02	Salt water	Salinity 6–8% from solar salterns, California	Rodriguez-Brito *et al.*, 2010	4440426.3
Medium salinity saltern	viral	33291	6	0.02	Salt water	Salinity 12–14% from solar salterns, California	Rodriguez-Brito *et al.*, 2010	4440427.3
Peru Margin 16mbsf	cellular	121414	18	0.01	Deep biosphere- marine sediment	Peru Margin ODP Leg 201 Site 1229, 16 meters below seafloor	Biddle *et al.*, 2008	4440973.3
Coral	cellular	1053275	144	0.01	Coral	*Porites compressa* coral samples collected at the Hawaii Institute for Marine Biology	Vega Thurber *et al.*, 2009	CAM_PROJ_CoralMetagenome
Tampa Bay	viral	257075	32	0.01	Fresh water	Prophages induced with mitomycin C from Tampa Bay water samples	McDaniel *et al.*, 2008	4440102.3
High salinity saltern	viral	136564	13	0.01	Salt water	Salinity 27–30% from solar salterns, California	Rodriguez-Brito *et al.*, 2010	4440421.3
Microbialites	cellular	257573	20	0.01	Micro-bialites	Highborne Cay, Bahamas	Breitbart *et al.*, 2009	4440061.3
Sargasso Sea	viral	399343	22	0.01	Open ocean	80 m, sampled June 2005, Sargasso Sea	Angly *et al.*, 2006	4441624.3

These include transposases, integrases, recombinases, and resolvases as defined by a keyword search in Pfam (database file included in supplementary material). Matches found using tblastn with a minimum e-value of 10–^5^. All metagenomes listed here were generated with shotgun pyrosequencing.

### Evidence of genomic plasticity in vent genomes

Having found evidence for gene transfer and viral integration in the genomes of vent cells and viruses, we sought evidence for either lysogenic virus integration or gene transfer events in other hydrothermal vent isolates. Of the 34 available fully sequenced vent genomes, 20 (59%) contain integrated viruses (Table S4). Most of these viruses encode capsid genes or genes such as DNA ligases, which have been identified before as being particularly abundant in the viral fraction of this hydrothermal vent sample [46]. However, identification of auxiliary metabolic genes (AMGs) in these viral genomes is difficult, partly due to the high abundance of unknown genes and partly because the boundaries between the viral and cellular genome are not always clearly delineated.

To better identify regions that have been transferred in vent genomes, we compared genomes from bacterial or archaeal isolates with sequences sampled directly from the environment. This strategy can identify potential hypervariable regions, or “genomic islands,” that display lower coverage than the rest of the genome [47]–[49]. Previous work with *Haloquadratum walsbyi* DSM 16790 [48] and *Prochlorococcus* genomes [47] used this technique to identify genomic islands that most likely represented regions of virally-mediated lateral gene transfer; in the case of *Prochlorococcus,* these genes are differentially expressed under light and nutrient stress [47].

**Figure 2:**
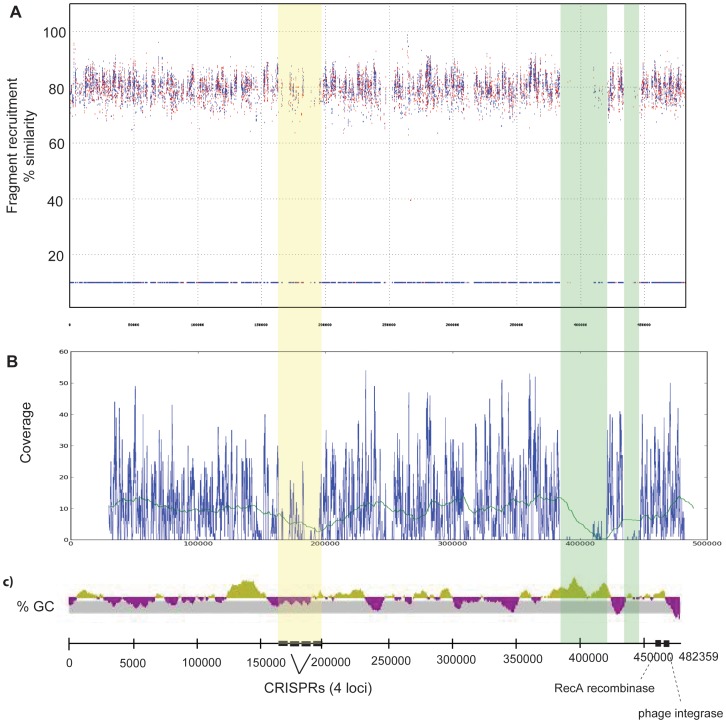
Recruitment plot of metagenomic reads to *Caminibacter mediatlanticus* TB-2. Cellular metagenomic reads were mapped to the longest contig of the draft genome of *C. mediatlanticus* TB-2, with percent similarity on the y-axis and base pair numbers on the x-axis (A). Coverage plot of read recruitment is shown per base pair, with blue line showing actual coverage and green line showing a convolution function of the coverage plot using a weighting of 50000 (B). Percent GC plot for the same contig is shown on the same scale, with base pair numbers marked below (C), and are annotated with CRISPR loci and recombinases or integrases found on the contig. Orange shading shows the location of CRISPR loci on the genome; green shading shows the location of two metagenomic islands.

We performed fragment recruitment of the hydrothermal vent cellular metagenome against the genomes of all isolates of hydrothermal vent bacteria and archaea available in the NCBI database. In most cases, the metagenome did not recruit to the isolate genomes with high enough coverage to yield useful data. *Nautilia profundicola* AmH successfully recruited reads at high coverage, but no metagenomic islands were found. Recruitment of the cellular metagenome to the longest contig (ABCJ01000001) of the draft genome of *Caminibacter mediatlanticus* TB-2, a chemolithotrophic, nitrate-ammonifying *Epsilonproteobacterium* that was isolated from the walls of a hydrothermal vent chimney on the Mid-Atlantic Ridge [50], yielded a number of regions with relatively low coverage (Figure 2). The first, a region with relatively low coverage between 160000 and 200000 bp, contains a series of CRISPR loci. CRISPR regions are dedicated to viral and plasmid immunity by effectively creating a library of previous infection, and therefore are highly specific to a given environment. Therefore recruitment to CRISPR loci should naturally yield lower coverage, particularly for a metagenome sampled in a different geographic location than this isolate. Aside from the CRISPR region, recruitment yielded two distinct genomic islands: one region of approximately 40 kbp, followed by a second low-coverage region of approximately 15 kbp (Figure 2), separated from each other by a 20 kbp region that includes a ribosome. The first genomic island coincides with a region with relatively high GC content, which suggests this region was transferred into the *C. mediatlanticus* genome. A phage integrase gene is located approximately 58 kbp downstream of the 3′ end of the first genomic island, though its presence there is not necessarily conclusive evidence that the region was introduced by a virus. The first genomic island begins near a tRNA gene, a common site for integration of horizontally transferred regions [51]. Many of the genes in both the first and second genomic islands encode proteins that interact with the environment, including sugar and nitrate membrane transporters, and proteins related to energy metabolism, including hydrogenases (Table S5). Possession of a diverse suite of hydrogenases can enhance metabolic flexibility in variable redox conditions, and has been observed in other *Epsilonproteobacteria* isolated from hydrothermal vents [52].

The presence of this hypervariable region indicates that genomic islands from genomes in vent environments encode genes related to environmental interactions and energy metabolism. The genomic island shown here is unique to a vent isolate from the Mid-Atlantic Ridge. The *C. mediatlanticus* strains present in our sample on the Juan de Fuca Ridge most likely have genomic islands of their own, though these cannot be identified without a fully sequenced strain from the Juan de Fuca Ridge. None of the sequenced strains from the Juan de Fuca Ridge had high enough coverage with our metagenome to identify genomic islands. However, the genomic island on *C. mediatlanticus* provides evidence of horizontal transfer of genes that facilitate metabolic flexibility in an environment that is very similar to the Juan de Fuca Ridge. From this we can hypothesize that genes related to environmental interactions and metabolic activity are transferred in our sample site.

### Quantification of relative gene enrichment in the viral fraction

**Figure 3:**
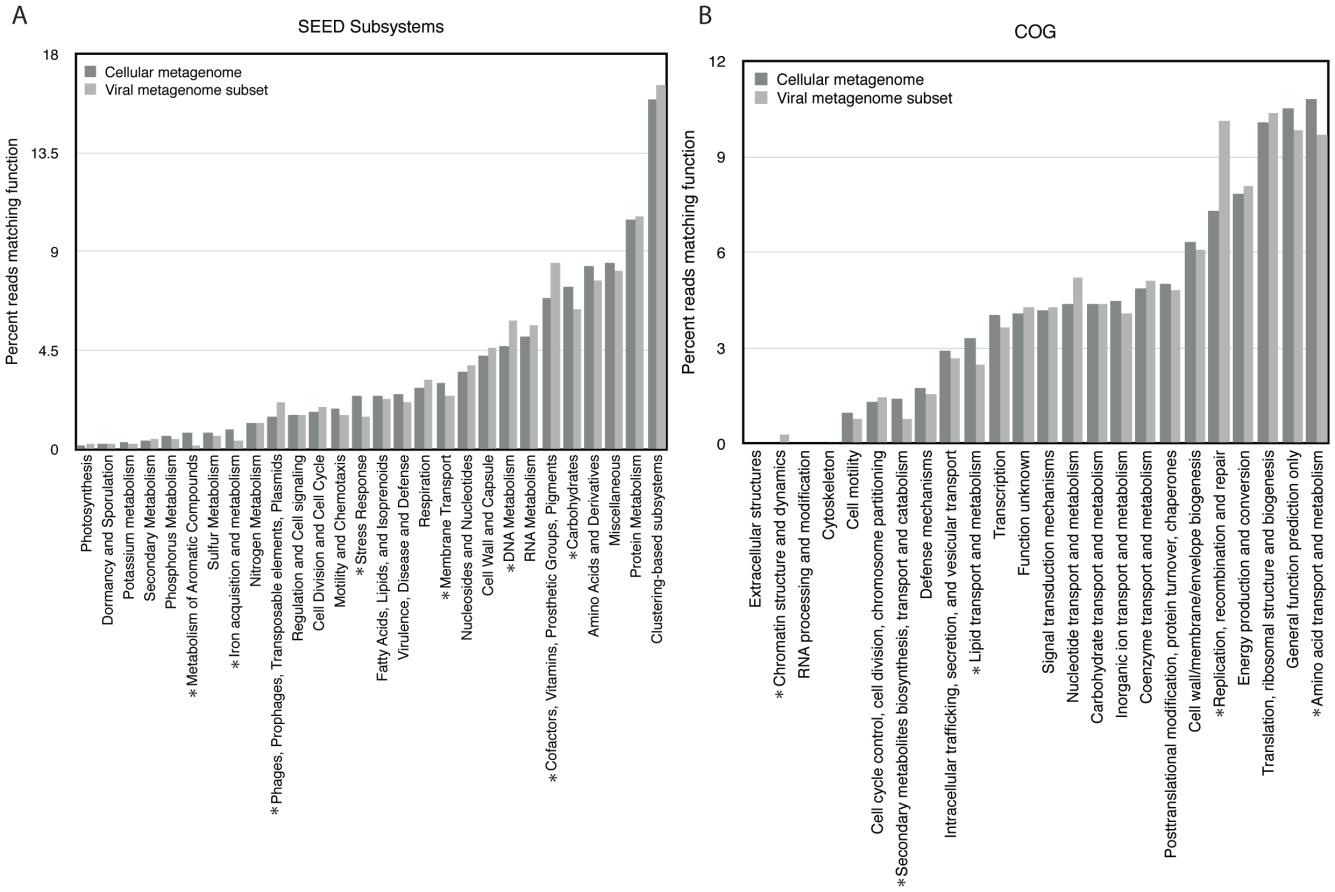
Functional comparisons of the hydrothermal vent cellular and viral subset metagenomes according to the SEED subsystems and Clusters of Orthologous Groups (COG) databases. Metagenomes were annotated in MG-RAST with a minimum e-value of 1e-03 and a minimum identity cutoff of 60%. A single asterisk indicates a significant difference in abundance between the viral subset and the cellular metagenome. A) Matches to the SEED subsystems database; B) matches to the COG database.

**Figure 4:**
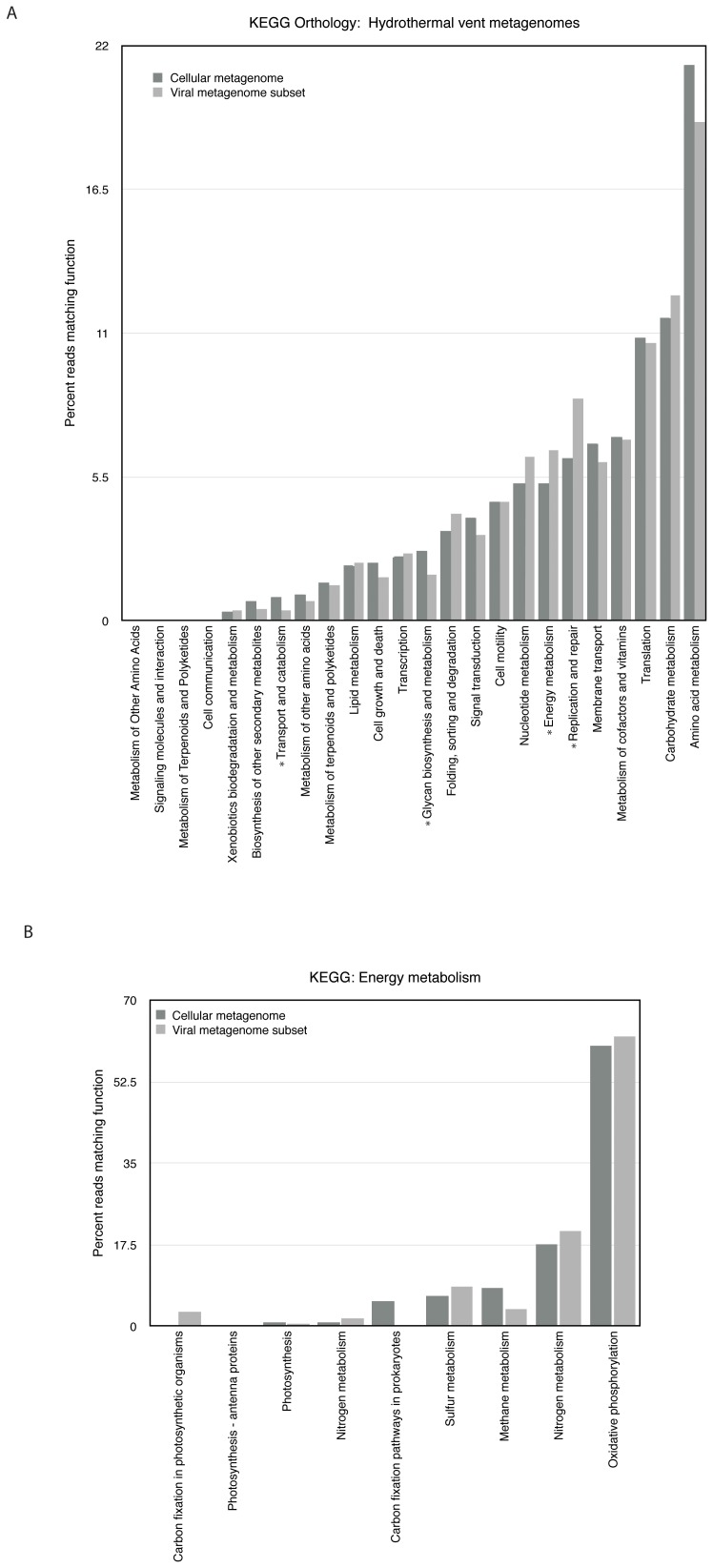
Functional comparisons of the hydrothermal vent cellular and viral subset metagenomes according to the KEGG Orthology annotation system. Metagenomes were annotated in MG-RAST with a minimum e-value of 1e-03 and a minimum identity cutoff of 60%. A single asterisk indicates a significant difference in abundance between the viral subset and the cellular metagenome. A) Matches to the KEGG Orthology database; B) Matches to the energy metabolism category of the KEGG Orthology database.

We next sought to determine whether there were differences in the relative enrichment of gene types in the cellular and viral fractions. In order to account for biases or omissions in various databases, we annotated reads in both the viral and cellular metagenomes according to three functional databases: the SEED Subsystems database [38], the Clusters of Orthologous Groups (COG) database [37], and the KEGG Orthology (KO) database [36] (Figures 3 and 4). Among all three databases, certain trends appeared. First, in all three cases the results indicated a high similarity in the overall relative abundances between functional genes in the cellular and viral fractions. A similar study of relative abundances of functional groups in viral and cellular metagenomes, conducted by Kristensen *et al.* [44] based on data collected by Dinsdale *et al.* [53], found a similar trend. While some of this is most likely due to cellular contamination in the viral fraction, as well as the presence of viruses in the cellular fraction, Kristensen *et al*. suggest this trend may also be due in part to the choice of available functional categories, which encompass functions that are generally cellular rather than viral. However, despite the likely presence of contamination in these datasets, direct comparison in this way enables us to compare the relative enrichment of certain types of genes in the viral and cellular gene pools. Comparisons between the viral subset and the cellular metagenome are shown in Figures 3 and 4; comparisons between the cellular metagenome, original virome, and viral subset are shown in Figure S3.

There was a statistically significant enrichment of reads matching phage, prophage, and transposable elements in the virome relative to the cellular metagenome (2.3% vs. 1.5%). These annotations were made according to the SEED Subsystems classification (Figure 3A), and we used Xipe-Totec [39] to assess statistical significance. Most viral gene hits were to distantly related head-tail viruses and cyanophage. The low percentage of matches to viral sequences overall is likely due in part to the relative lack of environmental viral sequences in public databases. We also observed an enrichment of reads matching cofactors, vitamins, prosthetic groups and pigments in the viral fraction (Figure 3A), which falls in line with studies identifying these types of genes on cyanophage genomes. Genes encoding vitamin B12 [53], [54], and the pigments psbA/D [55]–[58] and pebS [23] have previously been identified on cyanophage genomes. These virally encoded genes are thought to supplement host metabolism during infection, and similar processes may occur in this vent ecosystem.

We observed a statistically significant enrichment of reads matching amino acids and derivatives in the cellular fraction relative to the viral fraction, according to the COG (Figure 3B) and KO (Figure 4A) databases. These include genes related to the biosynthesis and metabolism of various amino acids, and are evidently not commonly carried on viral genomes in vent ecosystems, suggesting that viruses rely on their hosts for amino acid biosynthesis.

We also observed enrichment in reads related to replication, recombination and repair in the viral fraction, as annotated by both the COG and KO databases (Figure 3B, Figure 4A). These genes probably serve necessary functions in the synthesis and replication of viral DNA during the course of viral infection, and include genes like DNA and RNA polymerase, which are commonly encoded on viral genomes. The “replication and repair” category includes DNA ligases, which occur at very high abundances in the virome, as previously reported [46].

Finally, we also observed a statistically significant enrichment of genes related to energy metabolism as annotated by the KO database. Among reads annotated as matching the energy metabolism sub-category, 6.5% from the cellular metagenome and 8.4% from the viral subset were annotated as sulfur metabolism, whereas 8.2% of cellular versus 3.7% of viral metagenome reads annotated as belonging to methane metabolism (Figure 4B). The enrichment of energy metabolism genes corresponds with what we might expect given the observation of auxiliary metabolic genes (AMGs) in viral genomes from the surface oceans. A similar enrichment in energy metabolism genes was found in the viral fraction relative to the cellular fraction in samples from the Indian Ocean [59]. Similarly, photosynthesis genes encoded in cyanophage are known to be expressed during viral infection [20], [60], [61], and modeling work has indicated that these photosynthesis genes can enhance host fitness [62], [63]. For lytic viruses, energy metabolism genes may be expressed as a means to supplement host metabolism or to redirect resources for phage particle synthesis. Our analysis of fragment recruitment (Figure 2) indicated that genes related to energy metabolism had been successfully transferred between genomes in the past, and viruses are potential vectors for such gene transfer. One potential explanation for this enrichment, in line with the fragment recruitment results, is that highly abundant proviruses in the vent system express these genes while integrated in the host genome. By providing their hosts with new or supplemental means of surviving a challenging, dynamic environment, these proviruses boost host fitness, and in turn, enhance their own fitness.

To compare these results with other cellular-viral metagenome comparisons, we conducted an analysis of 20 cellular metagenomes and 23 viral metagenomes from other environments, all sequenced with 454 pyrosequencing and annotated with the KO database. Each of the cellular metagenomes had at least one viral counterpart sampled from the same environment. Metadata regarding these metagenomes are summarized in Table S6; the functional profiling analysis is depicted in Figure S4. The overall patterns of relative gene abundance between the viral and cellular fractions were similar to those observed in our sample. Specifically, in other environments functional annotations of viral and cellular metagenomic reads were strongly correlated, but viral metagenomes were more enriched in reads annotated as nucleotide metabolism and replication and repair. However, the relative enrichment of genes related to energy metabolism in the viral fraction, while apparent in the vent environment, was not a universal characteristic of viruses in other environments.

### Does selection operate differentially on viral and cellular genes?

Integral to the study of viral and cellular evolution is understanding how selection shapes the viral and cellular gene pools, and which genes are subject to stronger or weaker selection. Differing life strategies for cells and viruses, as well as disparate roles for functional genes within each of the respective gene pools, should leave different selective signatures on genes within each of these gene pools. To measure differential selection among viral and cellular genes, we calculated dN/dS ratios of genes encoded by the viral and cellular fractions. The challenge of calculating dN/dS ratios with shotgun metagenomic data is that the short sequences make it difficult to align long blocks of sequences to the same region of a gene. To circumvent this problem, we used the method developed by Tai *et al.* [42] to calculate dN/dS ratios from metagenomic reads, in which sequencing reads are mapped to the genomes of previously sequenced isolates. We used 80% identity as the threshold for mapping to genomes, a convention established previously [42]. While some work suggests that microbial “species” share an average nucleotide identity of 95% across the genome [64], tiling at that percentage did not yield enough hits for statistical analysis. Previous work has also indicated that below 80% identity, the number of reads recruited drops drastically, implying a biological threshold at 80% similarity [42]. These sequences therefore define the “population.” However, since the sequences used for analysis may be derived from multiple taxa, and we do not know the specific phylogenetic relationship of these sequences to each other, this method cannot determine which polymorphisms have become “fixed” in the population. Instead, this method provides an indicator of diversification within the environmental gene pool.

Both metagenomes were mapped to pre-existing hydrothermal vent isolates as a high-throughput means to align reads to many genes at once. The mapping analyses and calculation of dN/dS ratios are calculated by tiling metagenomic reads to the genomes of *Nautilia profundicola* AmH, *Thermococcus kodakarensis* KOD1, *Thermococcus onnurineus* NA1, *Caminibacter mediatlanticus* TB-2 (contig ABCJ01000001), and *Nitratiruptor* sp. SB155-2, which represent abundant strains in the vent environment. We attempted to map the virome to several existing viral genomes from various environments, but none exhibited sufficient depth of coverage to calculate dN/dS ratios, indicating that the genes encoded by viruses from this hydrothermal system are vastly different from those sequenced previously.

**Figure 5:**
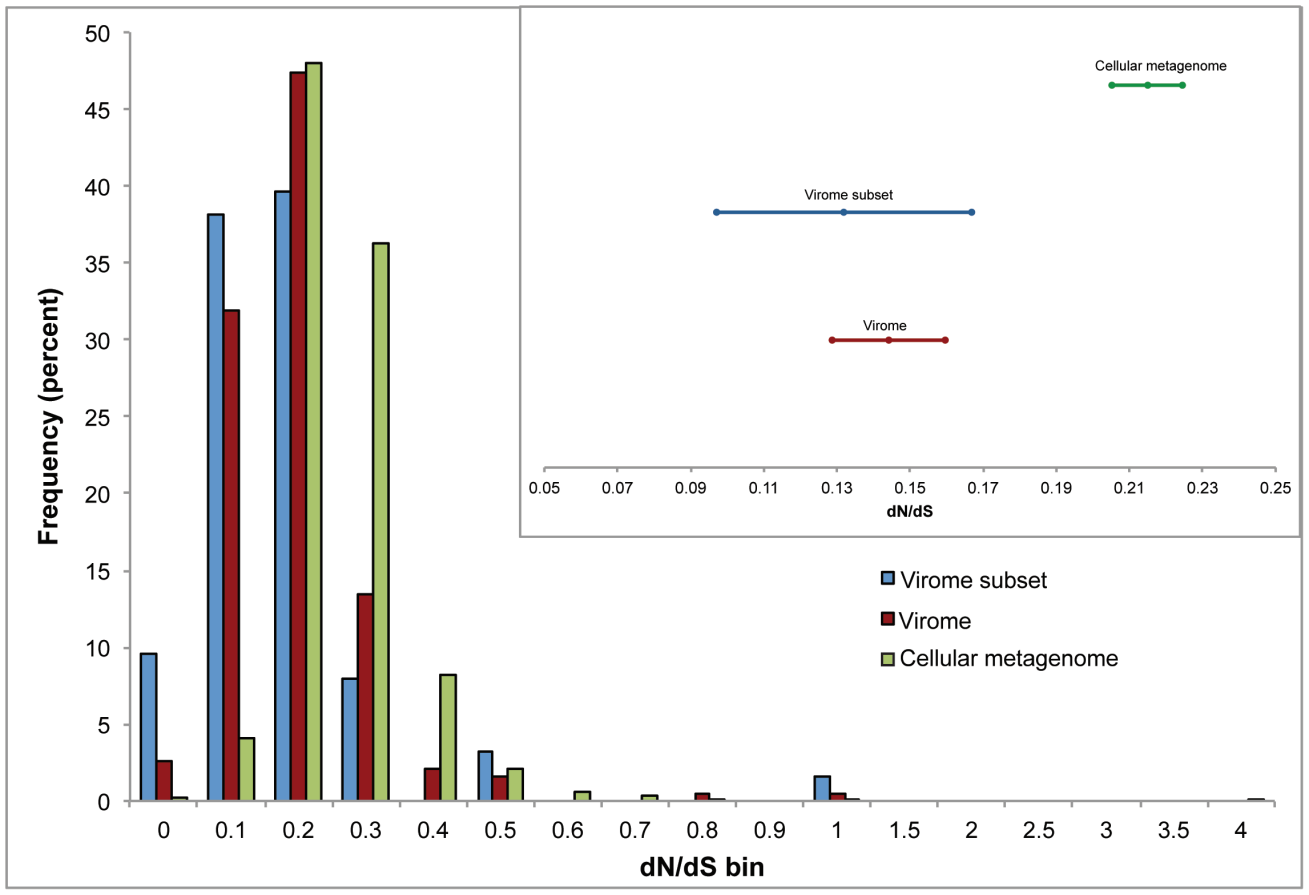
Histogram of dN/dS ratios for each metagenome. A total of 863 genes were included for the cellular metagenome calculation, 191 for the viral metagenome, and 64 for the viral subset. Values are shown only for genes that had a minimum depth coverage of 5 and minimum nucleotide coverage of 100. Frequency values are normalized by percent. Bins are scaled in increments of 0.1 until 1, and then in increments of 0.5. Inset shows mean and 95% confidence intervals for calculated dN/dS for all three data sets, indicating that the average cellular dN/dS is significantly greater than the average dN/dS for the viral metagenome.

Overall, the cellular metagenome mapped to 831 bacterial and 32 archaeal genes, with an average dN/dS of 0.22. This result indicates that genes encoded by cells in the vent environment are subject to purifying selection. The viral metagenome mapped to 85 bacterial and 106 archaeal genes, with an average dN/dS of 0.15, and the viral metagenome subset mapped to 39 bacterial and 25 archaeal genes, with an average dN/dS of 0.13 (Figure 5). These dN/dS values are significantly lower than the dN/dS of genes matching the cellular metagenome, within a confidence interval of 95%. This pattern was consistent for each of the genomes mapped (Figure S5). The viral and cellular metagenomes mapped to different genes in each of the strains listed above, and so slightly different sets of genes were used to make this calculation. However, the difference in overall dN/dS is not due solely to differences in the types of genes to which each metagenome mapped: when we examined the dN/dS for only the genes to which both metagenomes mapped, the calculated dN/dS was, on average, lower for the viral fraction compared to the cellular fraction (Figure S6). There were no clear trends in dN/dS for different gene categories (Figure S7).

These results indicate that both the viral and cellular fractions in this hydrothermal system are subject to purifying (negative) selection, but the viral gene pool is under stronger purifying selection than the cellular gene pool. One possible explanation for the overall difference in dN/dS between the viral and cellular gene pools is that very little variation in viral genes is permitted. In this scenario, viral genes are under such strong selection that deviations from the consensus protein sequence produce enough of a fitness difference to eliminate the viral mutant. The viral genes included in this analysis mapped to cellular genomes, and therefore are likely to be auxiliary metabolic genes carried on viral genomes, suggesting that AMGs carried by viruses are subject to purifying selection to a greater degree than their cellular counterparts.

This scenario becomes more complicated as a result of the relationship between virus and host. If a virus were primarily lytic, then a selective sweep could act directly on the viral particles, reducing phenotypic variation (and therefore nonsynonymous polymorphisms). If, however, a virus were primarily lysogenic, a larger proportion of time would be spent integrated in the genome of the host. In this situation, the selective sweeps would act on both host and virus, and we might not expect to see a significant difference in the dN/dS ratios between viral and cellular genes. The difference observed here may indicate that in this system, selection acts most strongly on viruses while they are in the process of replicating or when they are in virion form (free in the environment), and can therefore be selected separately from the host.

## Conclusions

The dynamic, recirculating conditions of sulfide-hosted hydrothermal vent systems, combined with the vast diversity of archaea, bacteria, and their accompanying viruses, create an ecosystem with high potential for widespread sharing of the communal gene pool. The unique sample analyzed here, which most likely pulled fluid both from high-temperature subsurface hydrothermal fluid and from cold background seawater, included microorganisms from a wide range of ecological habitats within the vent system. Having been sampled from the same point source, this implies that these diverse microorganisms have the potential to come into close contact in these vent systems. In doing so, there is potential for these microorganisms to exchange genes as well as viruses. Mobile elements were abundant in both the viral and cellular metagenomes we obtained, likely reflecting the abundance of lysogenic viruses, which require integrases for viral genome insertion into the host, as well as high potential for horizontal gene transfer. In the genomes of vent inhabitants, we found evidence for horizontal transfer of genes for environmental interaction and energy metabolism, suggesting selection for enhanced phenotypic plasticity. Moreover, a slight enrichment of genes related to processes such as cofactor synthesis and energy metabolism suggests that viruses in this hydrothermal system carry auxiliary metabolic genes.

Any genes found in the viral gene pool must have some utility in order to be retained on small viral genomes. The genes encoded on viral genomes may be used by lytic viruses as a means to facilitate the manufacture of viral particles, as has been observed in cyanophage previously. However, the prevalence of lysogenic viruses in vent habitats and the selective signatures observed here suggest that these vent viruses are selected to spend much of their time as integrated proviruses rather than as free virions. In this case, auxiliary metabolism genes may be expressed by integrated proviruses to benefit the host. Selection should favor traits by which proviruses boost host fitness while the fitness of the virus and host are intertwined. In turn, host cells benefiting from provirus-encoded genes may gain an adaptive advantage through enhanced metabolic flexibility, which may favor selection for cells harboring proviruses. This advantage complicates the symbiotic relationship between virus and host. While still capable of wreaking destruction upon the cells they infect, the data described here suggest a viral evolutionary strategy in which the virus-host relationship transcends from a parasitic relationship into a mutualistic one, as both host and virus seek to survive the dynamic, extreme environment in which they coexist.

## Supporting Information

Figure S1 Assignment of metagenomic contigs for the cellular metagenome (A) and the viral metagenome (B), based on di-, tri-, and tetranucleotide abundance determined by PhylopythiaS (McHardy et al., 2007). Boutique PhylopythiaS training datasets were created to classify contigs in the cellular and viral metagenomes as archaeal, bacterial, archaeal virus or bacterial virus.
(PDF)

Figure S2 Coverage and length of assembled contigs in the viral metagenome. Average coverage, shown on the x-axis, indicates the average coverage per base pair across the entire contig. Contig length is shown on the y-axis. Contigs are colored according to the assigned taxonomy.
(PDF)

Figure S3 These graphs accompany Figs. 3 and 4 in the main document, but include the original virome for comparison. One asterisk indicates a significant difference between the viral subset and the cellular metagenome; two asterisks indicate a significant difference between the original virome and the cellular metagenome.
(PDF)

Figure S4 Mean percentages of 20 cellular metagenomes and 23 viral metagenomes annotated according to the KEGG Orthology annotation system. Metagenomes were annotated in MG-RAST with a minimum e-value of 1e-03, minimum identity cutoff of 60%, and minimum alignment length of 15, and were derived from studies that directly compared viral and microbial metagenomes. Data from Dinsdale et al. 2008. Error bars indicate standard deviation of the mean.
(PDF)

Figure S5 Histograms of dN/dS for genes in three different genomes mapped by the cellular metagenome, virome, and virome subset. *Caminibacter mediatlanticus* TB-2 and *Nitratiruptor* sp. SB155-2 are not shown because the virome subset mapped only to four and zero genes in each of these genomes, respectively. Number of genes included in each histogram is indicated in parentheses.
(PDF)

Figure S6 Values of dN/dS for genes mapped by the virome versus dN/dS for the same genes mapped by cellular metagenome. The line has a slope of 1.
(PDF)

Figure S7 Box-and-whisker plots of dN/dS values for genes mapped each of the three metagenomic datasets. Genes are categorized according to KO annotation. A dotted line indicates where dN/dS = 1 and selection is neutral. Boxes indicate upper and lower quartiles; whiskers denote 1.5 times the interquartile range. Numbers below gene categories indicate the number of genes included for that category for the cellular and viral metagenomes, respectively.
(PDF)

Table S1 Summary of temperature and bacterial and viral counts from Hulk vent in the Main Endeavour Field. Temperature minimum was measured by temperature probes on a hydrothermal fluid sampler, temperature maximum was extrapolated based on dissolved silica concentrations.
(DOCX)

Table S2 List of viruses used to train PhylopythiaS for distinguishing between archaeal viruses and bacterial viruses.
(DOCX)

Table S3 Pfam domains included in search for genes associated with mobile genetic elements.
(DOCX)

Table S4 Numbers of proviruses identified in hydrothermal vent bacterial and archaeal genomes using Prophage Finder (Bose *et al*, 2006).
(DOCX)

Table S5 Annotation and best hit of reads within the low-coverage region of fragment recruitment from the Hulk cellular metagenome to the longest contig in the *Caminibacter mediatlanticus* TB-2 draft genome. See methods for details of fragment recruitment. Annotations are as listed by the draft annotation file released by the JCVI. Organism best hit determined by using blastn against the nr database.
(DOCX)

Table S6 List of viral and cellular metagenomes used for functional profiling of viral and cellular metagenomes using the KEGG Orthology database. Metagenomes obtained from the MG-RAST database were first analyzed by Dinsdale *et al.* (2009).
(DOCX)

File S1 Supplementary methods regarding the analysis of CRISPRs in the metagenomes.
(DOCX)
