# Supplementary material for: Evolutionary Strategies of Viruses, Bacteria and Archaea in Hydrothermal Vent Ecosystems Revealed through Metagenomics
Source: PLoS One. 2014 Oct 3;9(10):e109696. doi: 10.1371/journal.pone.0109696 (PMC4184897; doi:10.1371/journal.pone.0109696)
Supplement: Figure S1 — Assignment of metagenomic contigs for the cellular metagenome (A) and the viral metagenome (B), based on di-, tri-, and tetranucleotide abundance determined by PhylopythiaS (McHardy et al., 2007). Boutique PhylopythiaS training datasets were created to classify contigs in the cellular and viral metagenomes as archaeal, bacterial, archaeal virus or bacterial virus. (PDF) [file pone.0109696.s001.pdf]

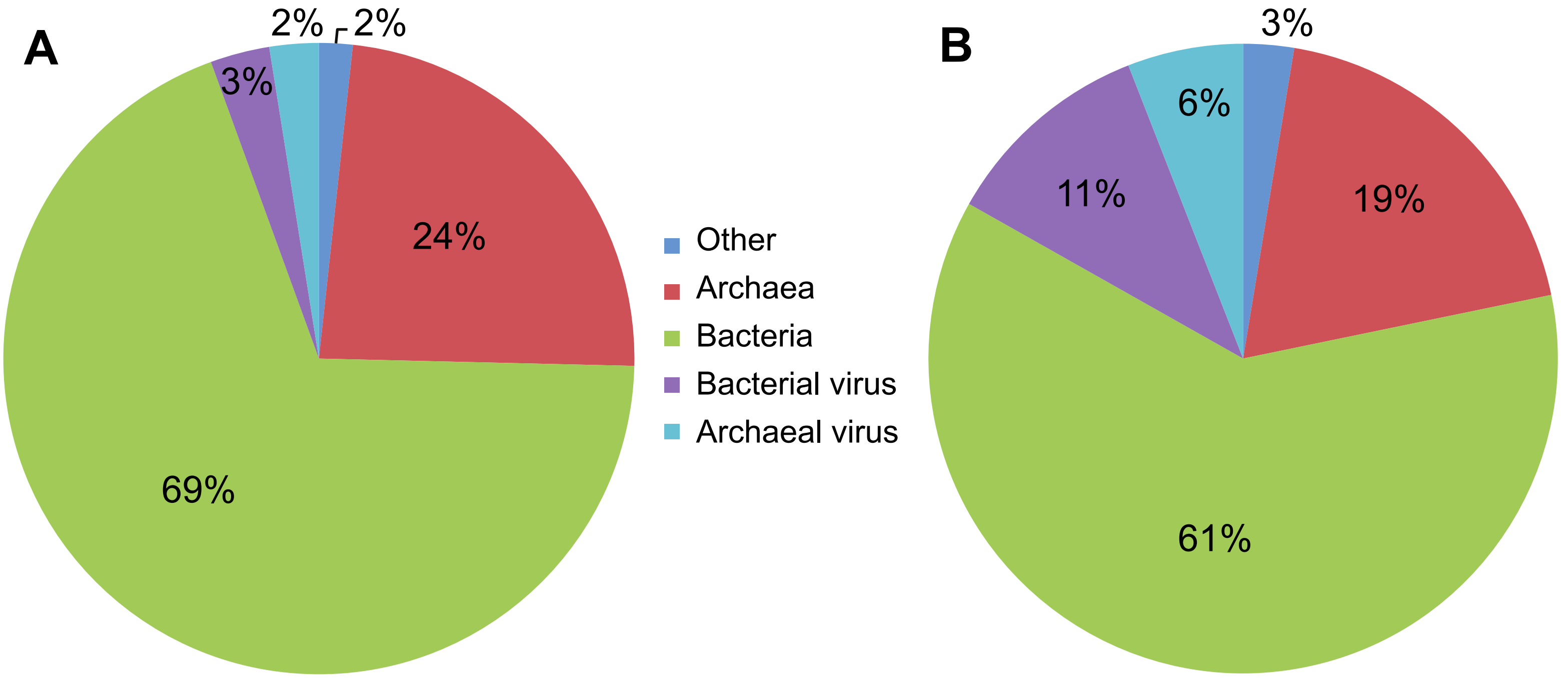

Figure S1. Assignment of metagenomic contigs based on di-, tri-, and tetranucleotide abundance as determined by PhylopythiaS (McHardy et al. 2007). Boutique PhylopythiaS training datasets were created to classify cotigs in the cellular and viral metagenomes as archaeal, bacterial, archaeal virus, or bacterial virus. A) Cellular metagenome contig classification. B) Viral metagenome contig classification.
