# Supplementary material for: Evolutionary Strategies of Viruses, Bacteria and Archaea in Hydrothermal Vent Ecosystems Revealed through Metagenomics
Source: PLoS One. 2014 Oct 3;9(10):e109696. doi: 10.1371/journal.pone.0109696 (PMC4184897; doi:10.1371/journal.pone.0109696)
Supplement: Figure S2 — Coverage and length of assembled contigs in the viral metagenome. Average coverage, shown on the x-axis, indicates the average coverage per base pair across the entire contig. Contig length is shown on the y-axis. Contigs are colored according to the assigned taxonomy. (PDF) [file pone.0109696.s002.pdf]

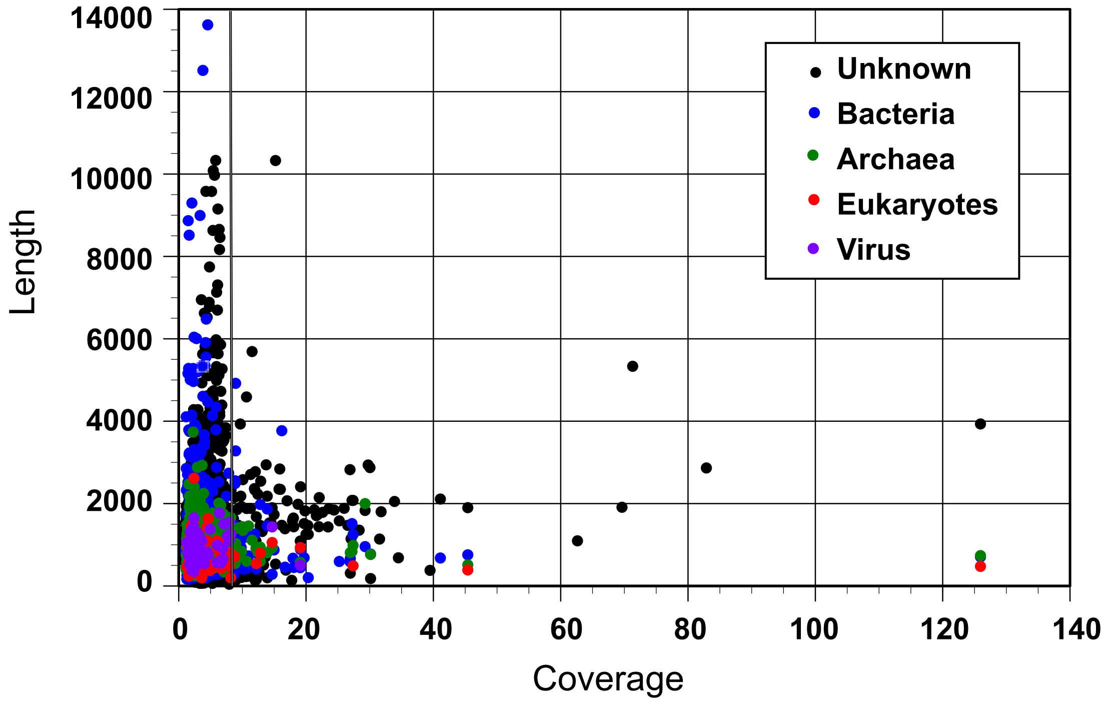

Figure S2. Coverage and length of assembled contigs in the viral metagenome. Average coverage, shown on the x-axis, indicates the average coverage per base pair across the entire contig. Contig length is shown on the y-axis. Contigs are colored according to the assigned taxonomy.
