# Supplementary material for: Evolutionary Strategies of Viruses, Bacteria and Archaea in Hydrothermal Vent Ecosystems Revealed through Metagenomics
Source: PLoS One. 2014 Oct 3;9(10):e109696. doi: 10.1371/journal.pone.0109696 (PMC4184897; doi:10.1371/journal.pone.0109696)
Supplement: Figure S3 — These graphs accompany Figs. 3 and 4 in the main document, but include the original virome for comparison. One asterisk indicates a significant difference between the viral subset and the cellular metagenome; two asterisks indicate a significant difference between the original virome and the cellular metagenome. (PDF) [file pone.0109696.s003.pdf]

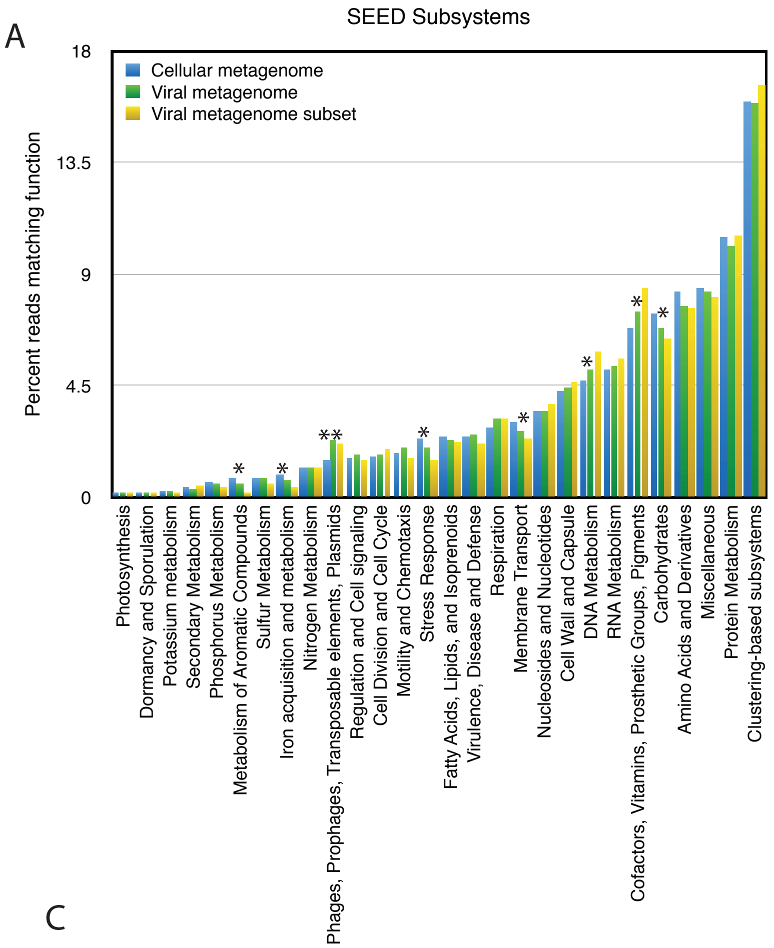

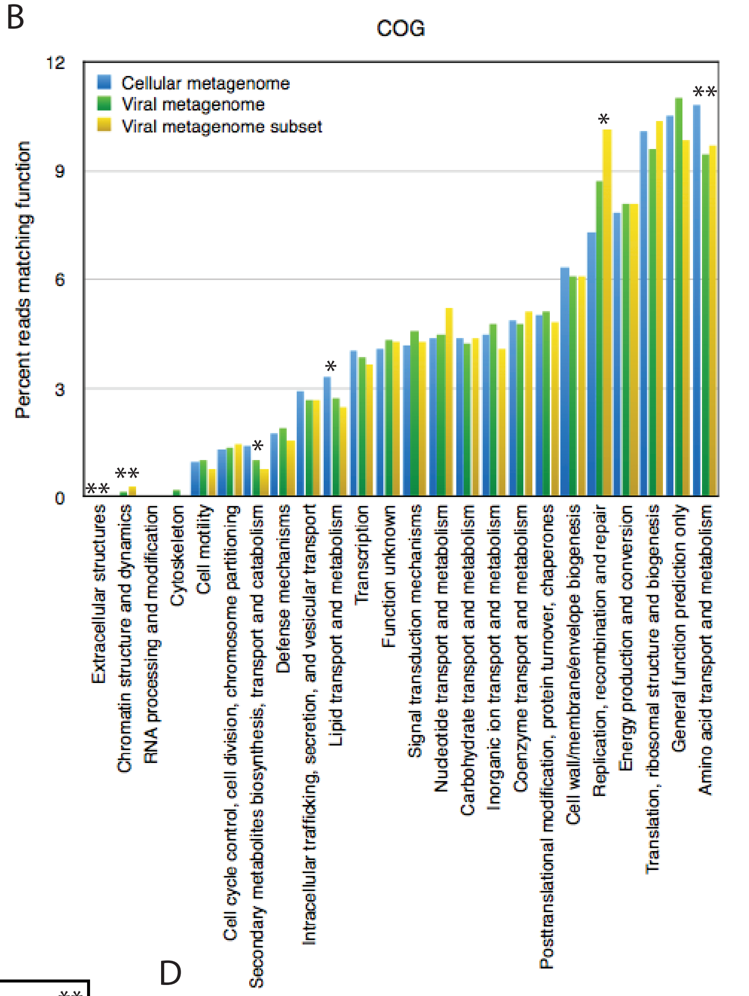

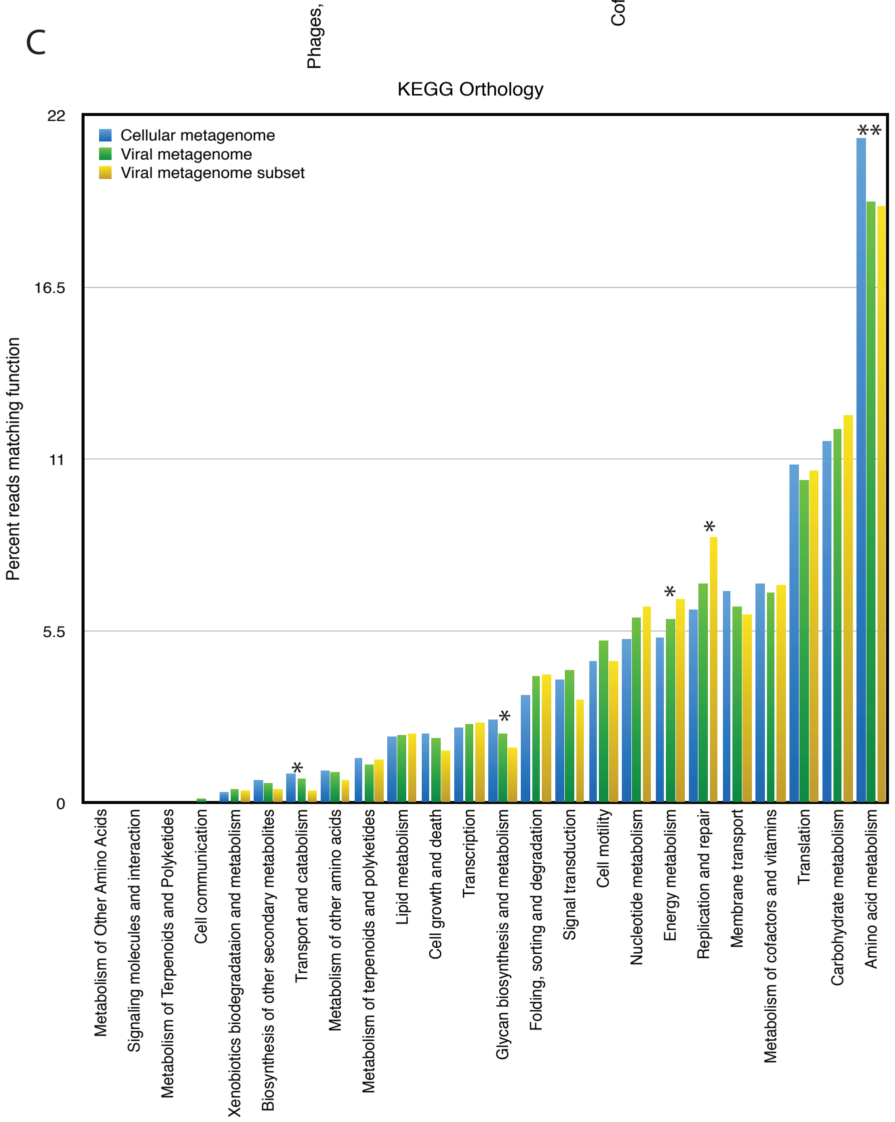

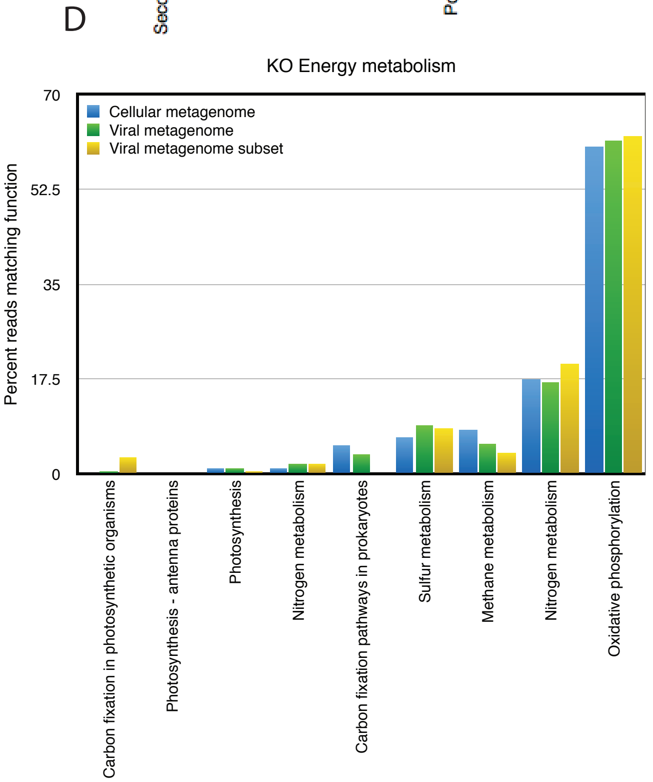

Figure S3. These graphs accompany Figs. 3 and 4 in the main document, but include the original virome for comparison. One asterisk indicates a significant difference between the viral subset and the cellular metagenome; two asterisks indicate a significant difference between the original virome and the cellular metagenome.
