# Supplementary material for: Evolutionary Strategies of Viruses, Bacteria and Archaea in Hydrothermal Vent Ecosystems Revealed through Metagenomics
Source: PLoS One. 2014 Oct 3;9(10):e109696. doi: 10.1371/journal.pone.0109696 (PMC4184897; doi:10.1371/journal.pone.0109696)
Supplement: Figure S4 — Mean percentages of 20 cellular metagenomes and 23 viral metagenomes annotated according to the KEGG Orthology annotation system. Metagenomes were annotated in MG-RAST with a minimum e-value of 1e-03, minimum identity cutoff of 60%, and minimum alignment length of 15, and were derived from studies that directly compared viral and microbial metagenomes. Data from Dinsdale et al. 2008. Error bars indicate standard deviation of the mean. (PDF) [file pone.0109696.s004.pdf]

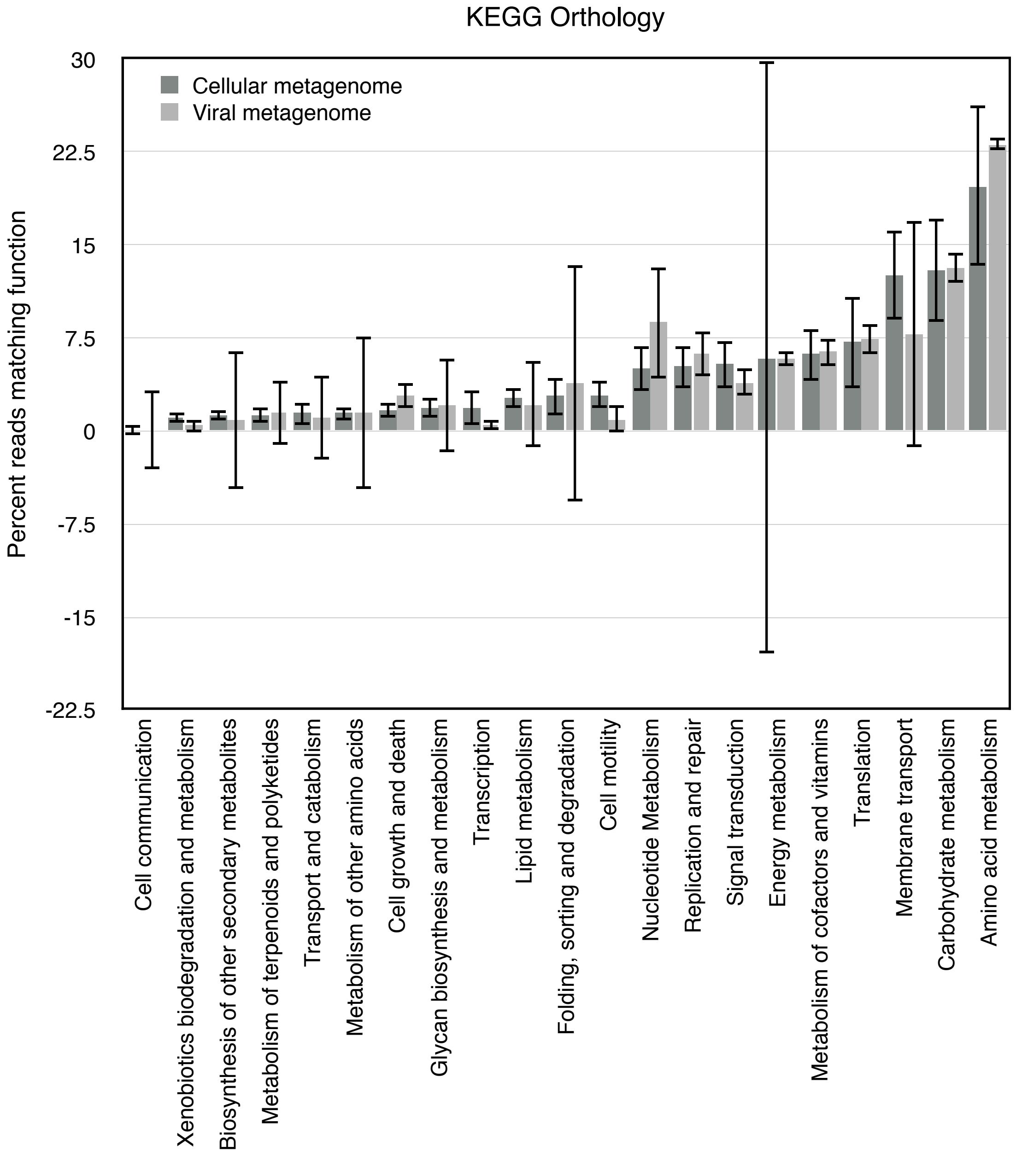

Figure S4. Mean percentages of 20 cellular metagenomes and 23 viral metagenomes annotated according to the KEGG Orthology annotation system. Metagenomes were annotated in MG-RAST with a minimum e-value of 1e-03, minimum identity cutoff of 60%, and minimum alignment length of 15, and were derived from studies that directly compared viral and microbial metagenomes. Data from Dinsdale et al. 2008. Error bars indicate standard deviation of the mean.
