# Supplementary material for: Evolutionary Strategies of Viruses, Bacteria and Archaea in Hydrothermal Vent Ecosystems Revealed through Metagenomics
Source: PLoS One. 2014 Oct 3;9(10):e109696. doi: 10.1371/journal.pone.0109696 (PMC4184897; doi:10.1371/journal.pone.0109696)
Supplement: Figure S5 — Histograms of dN/dS for genes in three different genomes mapped by the cellular metagenome, virome, and virome subset. Caminibacter mediatlanticus TB-2 and Nitratiruptor sp. SB155-2 are not shown because the virome subset mapped only to four and zero genes in each of these genomes, respectively. Number of genes included in each histogram is indicated in parentheses. (PDF) [file pone.0109696.s005.pdf]

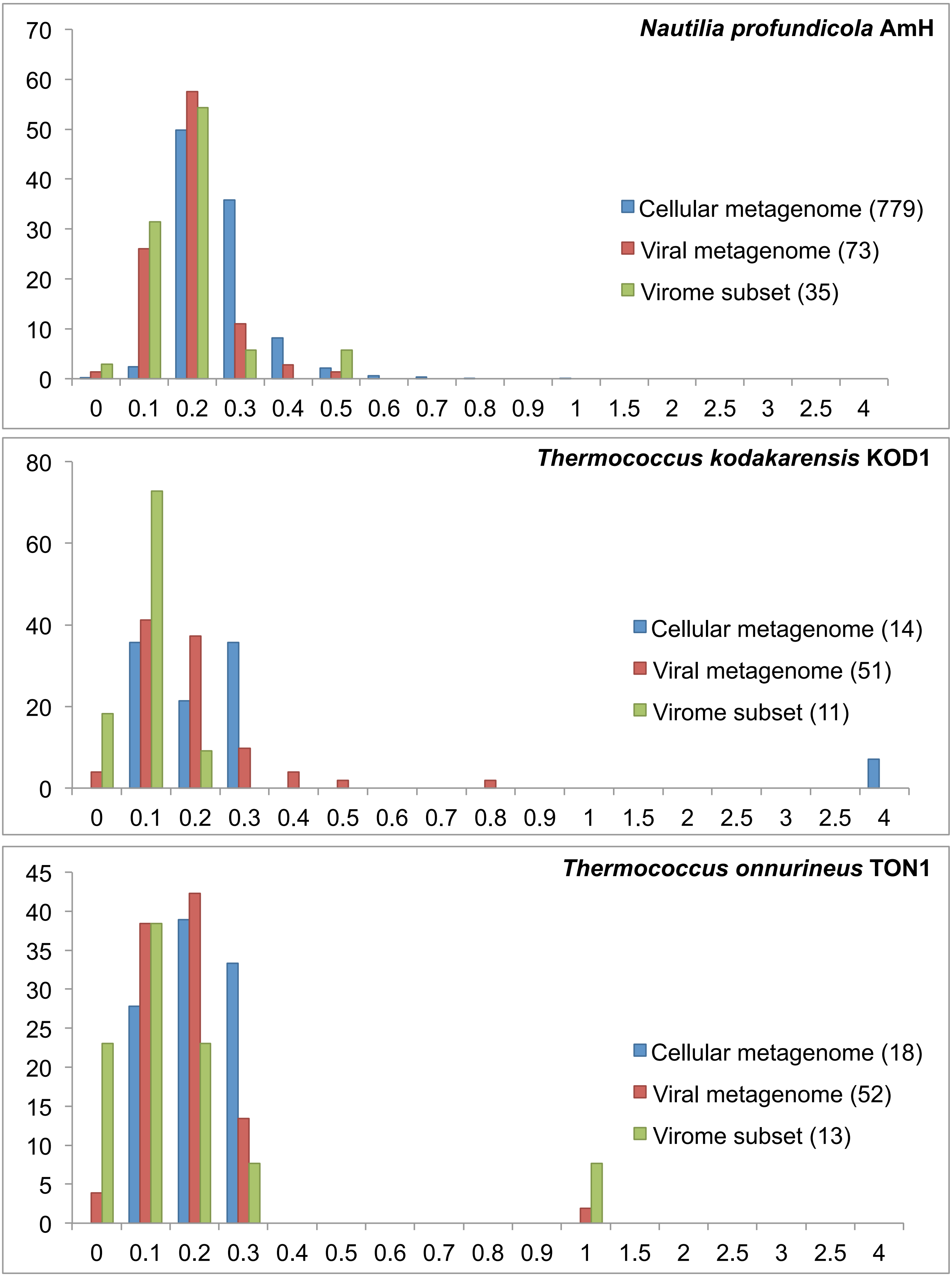

Figure S5. Histograms of dN/dS for genes in three different genomes mapped by the cellular metagenome, virome, and virome subset. *Caminibacter mediatlanticus* TB-2 and *Nitratiruptor* sp. SB155-2 are not shown because the virome subset mapped to only four and zero genes in each of these genomes, respectively. Numbers in legends indicate the number of genes included in each histogram.
