# Supplementary material for: Evolutionary Strategies of Viruses, Bacteria and Archaea in Hydrothermal Vent Ecosystems Revealed through Metagenomics
Source: PLoS One. 2014 Oct 3;9(10):e109696. doi: 10.1371/journal.pone.0109696 (PMC4184897; doi:10.1371/journal.pone.0109696)
Supplement: Figure S6 — Values of dN/dS for genes mapped by the virome versus dN/dS for the same genes mapped by cellular metagenome. The line has a slope of 1. (PDF) [file pone.0109696.s006.pdf]

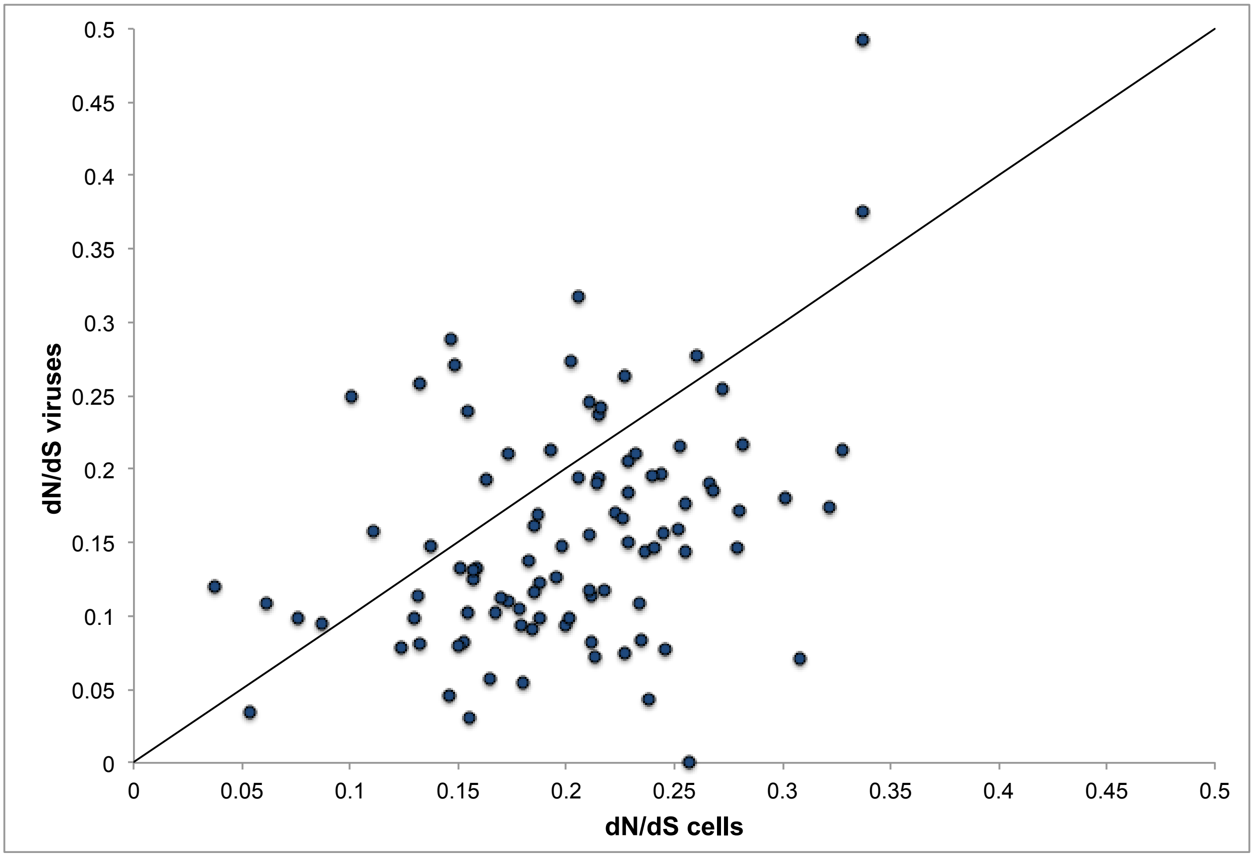

Figure S6. dN/dS for genes mapped by the viral metagenome versus dN/dS for the same genes mapped by the cellular metagenome. The line has a slope of 1.
