# Supplementary material for: Evolutionary Strategies of Viruses, Bacteria and Archaea in Hydrothermal Vent Ecosystems Revealed through Metagenomics
Source: PLoS One. 2014 Oct 3;9(10):e109696. doi: 10.1371/journal.pone.0109696 (PMC4184897; doi:10.1371/journal.pone.0109696)
Supplement: Figure S7 — Box-and-whisker plots of dN/dS values for genes mapped each of the three metagenomic datasets. Genes are categorized according to KO annotation. A dotted line indicates where dN/dS = 1 and selection is neutral. Boxes indicate upper and lower quartiles; whiskers denote 1.5 times the interquartile range. Numbers below gene categories indicate the number of genes included for that category for the cellular and viral metagenomes, respectively. (PDF) [file pone.0109696.s007.pdf]

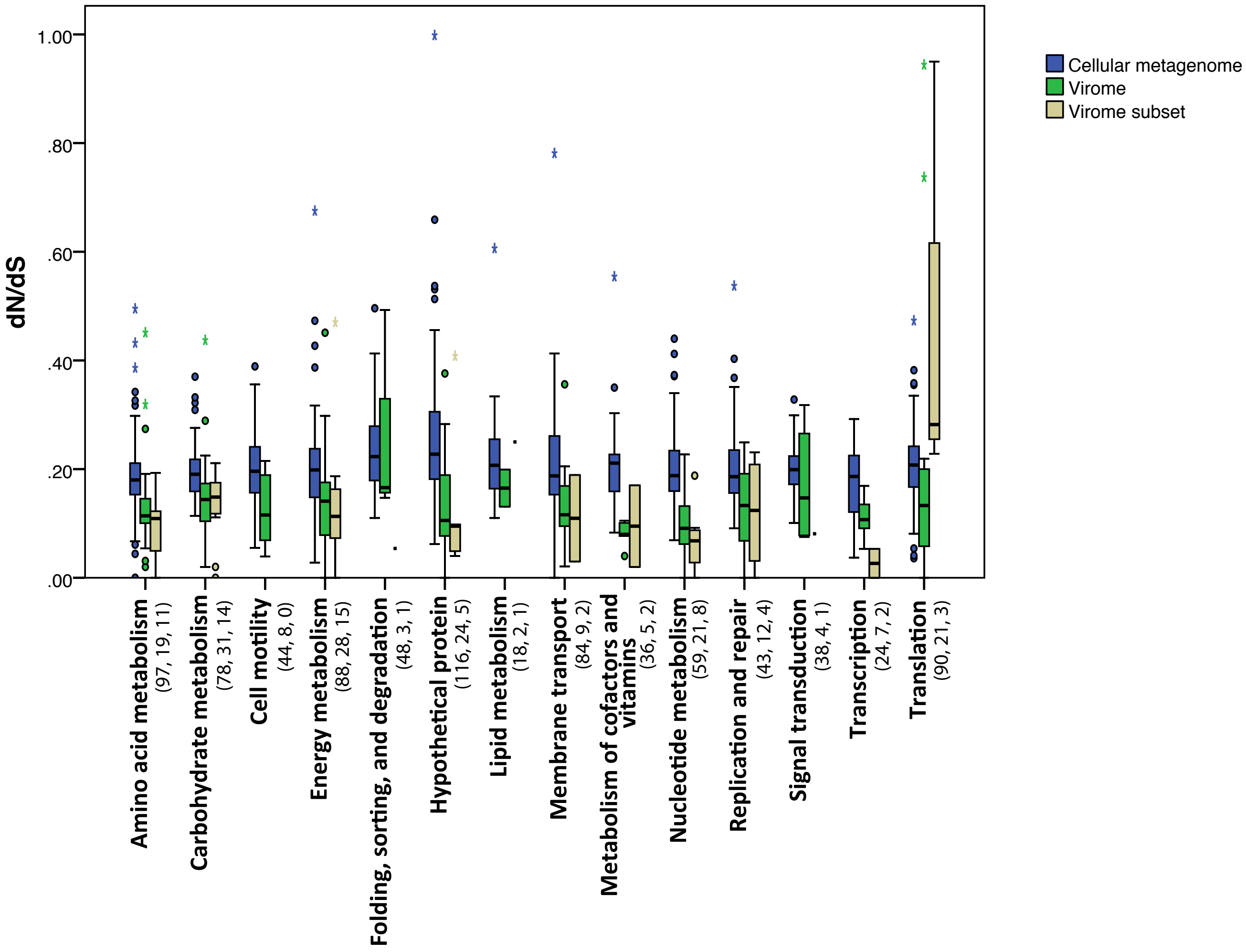


**Figure S7**. Box-and-whisker plots of dN/dS values for genes mapped by each of the three metagenomic datasets. Genes are categorized according to KO annotation. A dotted line indicates where dN/dS = 1 and selection is neutral. Boxes indicate upper and lower quartiles, and whiskers denote 1.5 times the interquartile range. Numbers below gene categories indicate the number of genes included for that category for the cellular metagenome, viral metagenome, and virome subset, respectively.
