# Supplementary material for: Evolutionary Strategies of Viruses, Bacteria and Archaea in Hydrothermal Vent Ecosystems Revealed through Metagenomics
Source: PLoS One. 2014 Oct 3;9(10):e109696. doi: 10.1371/journal.pone.0109696 (PMC4184897; doi:10.1371/journal.pone.0109696)
Supplement: Table S1 — Summary of temperature and bacterial and viral counts from Hulk vent in the Main Endeavour Field. Temperature minimum was measured by temperature probes on a hydrothermal fluid sampler, temperature maximum was extrapolated based on dissolved silica concentrations. (DOCX) [file pone.0109696.s008.docx]

**Table S1**. Summary of temperature and bacterial and viral counts from Hulk vent in the Main Endeavour Field. Temperature minimum was measured by temperature probes on a hydrothermal fluid sampler, temperature maximum was extrapolated based on dissolved silica concentrations.

| **Physical characteristic** | **Value** |
| --- | --- |
| Temperature | 13–130˚C |
| Bacterial counts | 1.69 x 10^7^ cells/ml^1^ |
| Viral counts | 6.80 x 10^6^ VLPs/ml^2^ |

^1^ Cell counts prior to filtration through the Steripak filter unit. ^2^ Viral-like-particle (VLP) counts after filtration through the Steripak filter unit. VLPs were not reliably countable prior to filtration due to the abundance of biomass and exopolysaccharide material in the sample.
