# Supplementary material for: Evolutionary Strategies of Viruses, Bacteria and Archaea in Hydrothermal Vent Ecosystems Revealed through Metagenomics
Source: PLoS One. 2014 Oct 3;9(10):e109696. doi: 10.1371/journal.pone.0109696 (PMC4184897; doi:10.1371/journal.pone.0109696)
Supplement: Table S2 — List of viruses used to train PhylopythiaS for distinguishing between archaeal viruses and bacterial viruses. (DOCX) [file pone.0109696.s009.docx]

**Table S2**. List of viruses used to train PhylophythiaS for distinguishing between archaeal viruses and bacterial viruses.

| **Archaeal viruses** | **Bacterial viruses** |
| --- | --- |
| *Acidianus* bottle-shaped virus | *Acinetobacter* phage 133 |
| *Acidianus* spindle-shaped virus 1 | Bacteriophage 11b |
| Haloarcula hispanica pleomorphic virus 1 | Bacteriophage Aeh1 |
| *Halorubrum* phage HF2 | Bacteriophage T3 complete genome strain Luria |
| His1 virus | *Burkholderia* phage phi644-2 |
| His2 virus | *Campylobacter* phage CP220 |
| Hyperthermophilic Archaeal Virus 1 | Deep-sea thermophilic phage D6E |
| Hyperthermophilic Archaeal Virus 2 | *Methanothermobacter* prophage psiM100 |
| *Pyrococcus abyssi* virus 1 | *Ostreococcus tauri* virus 1 |
| *Sulfolobus islandicus* rod-shaped virus 2 | *Prochlorococcus* phage P-SSM2 |
| *Sulfolobus islandicus rudivirus* 1 variant XX | *Pseudomonas* phage 201phi2-1 |
| *Sulfolobus* turreted icosahedral virus 2 | *Pseudomonas* phage gh-1 |
| *Sulfolobus* virus Kamchatka 1 | *Synechococcus* phage S-PM2 |
| *Thermoproteus tenax* spherical virus 1 | *Thermus* phage IN93 |
|  | *Thermus* phage P23-45 |
|  | *Thermus* phage P23-77 |
|  | *Thermus* phage P74-26 |
|  | *Thermus* phage phiYS40 |
|  | *Vibrio* phage ICP1_2004_A |
|  | Vibriophage VP4 |
