# Supplementary material for: Evolutionary Strategies of Viruses, Bacteria and Archaea in Hydrothermal Vent Ecosystems Revealed through Metagenomics
Source: PLoS One. 2014 Oct 3;9(10):e109696. doi: 10.1371/journal.pone.0109696 (PMC4184897; doi:10.1371/journal.pone.0109696)
Supplement: Table S3 — Pfam domains included in search for genes associated with mobile genetic elements. (DOCX) [file pone.0109696.s010.docx]

**Table S3.** Pfam domains included in search for genes associated with mobile genetic elements.

| **Pfam Accession** | **Description** |
| --- | --- |
| PF00552 | Integrase DNA binding domain |
| PF00665 | rve Integrase |
| PF01609 | Transposase DDE domain |
| PF01797 | Transposase IS200 like |
| PF09299 | Mu transposase C-terminal |
| PF00154 | recA bacterial DNA recombination protein |
| PF00239 | Resolvase |
| PF00589 | phage integrase |
| PF00872 | Transposase, Mutator family |
| PF01076 | Plasmid recombination enzyme |
| PF01385 | Probable transposase |
| PF01526 | Tn3 transposase DDE domain |
| PF01527 | Helix-turn-helix transposase |
| PF01548 | Transposase |
| PF01610 | Transposase |
| PF01710 | Transposase helix-turn-helix |
| PF02022 | integrase zinc binding domain |
| PF02281 | Transposase Tn5 dimerisation domain |
| PF02316 | Mu DNA-binding domain |
| PF02371 | Transposase IS116/IS110/IS902 family |
| PF02534 | Type IV secretory system conjugative DNA transfer |
| PF02646 | RmuC family- DN recombination proteins |
| PF02899 | phage integrase, N-terminal SAM-like domain |
| PF02914 | Bacteriophage Mu transposase |
| PF02920 | DNA binding domain of tn916 integrase |
| PF02945 | Recombination endonucelase VII |
| PF03050 | Transposase IS66 family |
| PF03400 | IS1 transposase |
| PF03837 | RecT family, involved in recombination |
| PF03838 | Recombination protein U |
| PF03930 | Recombinase Flp protein N-terminal domain |
| PF04404 | ERF superfamily-- recombination proteins |
| PF04693 | Archaeal putative transposase ISC1217 |
| PF04740 | LXG domain of WXG superfamily- not sure why this was included. |
| PF04754 | Putative transposase, YhgA-like |
| PF04986 | Putative transposase |
| PF05202 | Recombinase Flp protein |
| PF05598 | transposase domain |
| PF05717 | IS66 Orf2 like protein (essential for transposition) |
| PF07508 | Recombinase |
| PF07592 | Rhodopiruellula transposase DDE domain |
| PF08423 | Rad51-- DNA repair and recombination protein |
| PF09003 | bacteriophage lambda integrase, N-terminal domain |
| PF09034 | Excisionase from transposon Tn916 |
| PF09124 | T4 recombination endonuclease VII, dimerisation |
| PF09588 | YqaJ-like viral recombinase domain |
| PF10136 | Site-specific recombinase |
| PF10551 | MULE transposase domain |
| PF12834 | Phage integrase, N-terminal |
| PF12835 | Integrase_1 |
| PF12940 | Recombination-activation protein 1 (RAG1) |
| PF13009 | Putative phage integrase |
| PF13408 | Recombinase zinc beta ribbon |
| PF13495 | Phage integrase, N-terminal SAM-like domain |
| PF13542 | Helix-turn-helix domain of transposase family ISL3 |
| PF13683 | Integrase core domain |
| PF13751 | Transposase DDE domain |
