# Supplementary material for: Evolutionary Strategies of Viruses, Bacteria and Archaea in Hydrothermal Vent Ecosystems Revealed through Metagenomics
Source: PLoS One. 2014 Oct 3;9(10):e109696. doi: 10.1371/journal.pone.0109696 (PMC4184897; doi:10.1371/journal.pone.0109696)
Supplement: Table S4 — Numbers of proviruses identified in hydrothermal vent bacterial and archaeal genomes using Prophage Finder (Bose et al, 2006). (DOCX) [file pone.0109696.s011.docx]

**Table S4.** Numbers of proviruses identified in hydrothermal vent bacterial and archaeal genomes using Prophage Finder (Bose *et al*, 2006).

| **Organism** | **Number of predicted prophage** |
| --- | --- |
| *Aciduliprofundum boonei* T469 | 2 |
| *Archaeoglobus profundus* DSM 5631 | 2 |
| *Archaeoglobus fulgidus* DSM 4304 | 1 |
| *Aquifex aeolicus* VF5 | 1 |
| *Caminibacter mediatlanticus* TB2 | 0 |
| *Deferribacter desulfuricans* SSM | 1 |
| *Ferroglobus placidus* DSM 10642 | 1 |
| *Hyperthermus butylicus* DSM 5456 | 0 |
| *Ignicoccus hospitalis* KIN4/I | 0 |
| *Methanocaldococcus* sp. FS406-22 | 0 |
| *Methanocaldococcus fervens* AG86 | 0 |
| *Methanocaldococcus jannaschii* DSM 2661 | 0 |
| *Methanocaldococcus vulcanius* M7 | 0 |
| *Methanopyrus kandleri* AV19 | 0 |
| *Nanoarchaeum equitans* Kin4-M | 0 |
| *Nautilia profundicola* AmH | 1 |
| *Nitratiruptor* sp. SB155-2 | 4 |
| *Persephonella marina* EX-H1 | 4 |
| *Pyrobaculum aerophilum* str. IM2 | 0 |
| *Pyrococcus abyssi* GE5 | 1 |
| *Pyrococcus furiosus* DSM 3638 | 1 |
| *Pyrococcus horikoshii* OT3 | 2 |
| *Pyrococcus* sp NA2 | 2 |
| *Pyrococcus* sp. ST04 | 0 |
| *Rhodothermus marinus* DSM 4252 | 1 |
| *Staphylothermus marinus* F1 | 0 |
| *Thermus thermophilus* HB8 | 2 |
| *Thermococcus gammatolerans* EJ3 | 0 |
| *Thermococcus kodakarensis* KOD1 | 0 |
| *Thermococcus onnurineus* NA1 | 2 |
| *Thermococcus sibricus* MM_739 | 1 |
| *Thermococcus* sp. CL1 | 2 |
| *Thermotoga neapolitana* DSM 4359 | 1 |
| *Thiomicrospira crunognea* XCL 2 | 4 |
