# Supplementary material for: Evolutionary Strategies of Viruses, Bacteria and Archaea in Hydrothermal Vent Ecosystems Revealed through Metagenomics
Source: PLoS One. 2014 Oct 3;9(10):e109696. doi: 10.1371/journal.pone.0109696 (PMC4184897; doi:10.1371/journal.pone.0109696)
Supplement: Table S5 — Annotation and best hit of reads within the low-coverage region of fragment recruitment from the Hulk cellular metagenome to the longest contig in the Caminibacter mediatlanticus TB-2 draft genome. See methods for details of fragment recruitment. Annotations are as listed by the draft annotation file released by the JCVI. Organism best hit determined by using blastn against the nr database. (DOCX) [file pone.0109696.s012.docx]

**Table S5**. Annotation and best hit of reads within the low-coverage region of fragment recruitment from the Hulk cellular metagenome to the longest contig in the *Caminibacter mediatlanticus* TB-2 draft genome. See methods for details of fragment recruitment. Annotations are as listed by the draft annotation file released by the JCVI. Organism best hit determined by using blastn against the nr database.

| **Basepairs** | **Annotation** | **Organism best hit** |
| --- | --- | --- |
| 358816-386118 | ABC-type sugar transport system, permease component | Nautilia profundicola AmH |
| 386196-387833 | ATPase involved in DNA repair | Nautilia profundicola AmH |
| 387830-388561 | Chromosome segregation ATPases | Nautilia profundicola AmH |
| 3888827-389372 | Anaerobic dehydrogenases, typical selenocysteine-containing | Nautilia profundicola AmH |
| 389421-391646 | Anaerobic dehyrogenases, typical selenocysteine-containing | Nautilia profundicola AmH |
| 391657-392250 | Fe-S-cluster-containing hydrogenase components I | Arcobacter nitrofigilis DSM7299 |
| 392228-393193 | Cytochrome b subunit of formate dehydrogenase | Nautilia profundicola AmH |
| 393295-394212 | ABC-type molybdate transport system, periplasmic component | Nautilia profundicola AmH |
| 394212-395165 | ABC-type sugar transport systems, ATPase components | Deferribacter desulfuricans SSM1 |
| 395174-395899 | ABC-type sulfate transport system, permease component | Nautilia profundicola AmH |
| 396139-397005 | Formate/nitrite family of transporters | Desulfurobacterium thermolithotrophicum DSM 11699 |
| 397286-397708 | Ni,Fe-hydrogenase maturation factor | No closely related hits |
| 397705-398049 | ABC-type cobalt transport system, ATPase component | No closely related hits |
| 398042-398884 | Ni,Fe-hydrogenase III small subunit | Arcobacter nitrofigilis DSM7299 |
| 398881-399420 | Formate hydrogenlyase subunit 6/NADH:ubiquinone oxidoreductase | Arcobacter nitrofigilis DSM7299 |
| 399430-401169 | Ni,Fe-hydrogenase III component G | Arcobacter nitrofigilis DSM7299 |
| 401180-402661 | Formate hydrogenlyase subunit 3/Multisubunit Na+/H+ antiporter, MhhD subunit | Arcobacter nitrofigilis DSM7299 |
| 402665-403279 | Hydrogenase 4 membrane component | Arcobacter nitrofigilis DSM7299 |
| 403320-404246 | Formate hydrogenlyase subunit 4 | Arcobacter nitrofigilis DSM7299 |
| 404257-406224 | Formate hydrogenlyase subunit 3/Multisubunit Na+/H+ antiporter, MhhD subunit | Arcobacter nitrofigilis DSM7299 |
| 406235-406798 | Fe-S-cluster-containing hydrogenase components 2 | Arcobacter nitrofigilis DSM7299 |
| 406801-408564 | Anaerobic dehydrogenase, typically selenocysteine-containing | Desulfurobacterium thermolithotrophicum DSM 11699 |
| 408613-409032 | Anaerobic dehydrogenases, typically selenocysteine-containing | Desulfurobacterium thermolithotrophicum DSM 11699 |
| 409198-409839 | camp-binding proteins- catabolite gene activator and regulatory subunit of camp-dependent protein kinases | No close matches. |
| 409895-411214 | Selenocysteine synthase [seryl-tRNASer selenium transferase) | Arcobacter butzleri RM4018 |
| 411211-413034 | Selenocysteine-specific translation elongation factor | Nautilia profundicola AmH |
| 413355-413939 | Predicted redox protein, regulator of disulfide bond formation | Nautilia profundicola AmH/Campylobacter lari RM2100 |
| 413949-415064 | Transglutaminase-like enzymes, putative cysteine proteases | Nautilia profundicola AmH |
| 415068-415655 | Predicted redox protein, regulator of disulfide bond formation | Nautilia profundicola AmH |
| 415657-415929 | Cation transport ATPase | No closely related hits |
| 415926-416282 | Uncharacterized conserved protein involved in intracellular sulfur reduction | Nautilia profundicola AmH |
| 416348-417325 | Selenophosphate synthase | Nautilia profundicola AmH |
| 417332-420496 | Predicted phosphohydrolases | Thermodesulfatator indicus DSM 15286 |
| High coverage region between genomic islands 1 and 2 | | |
| 440514-442073 | Transcriptional regulator | No close matches |
| 442082-443608 | Glycosyltransferases, probably involved in cell wall biogenesis | Nitratiruptor sp. SB155-2 |
| 443626-444942 | Predicted UDP-glucose 6-dehydrogenase | Nitratiruptor sp. SB155-2 |
