# Supplementary material for: Evolutionary Strategies of Viruses, Bacteria and Archaea in Hydrothermal Vent Ecosystems Revealed through Metagenomics
Source: PLoS One. 2014 Oct 3;9(10):e109696. doi: 10.1371/journal.pone.0109696 (PMC4184897; doi:10.1371/journal.pone.0109696)
Supplement: Table S6 — List of viral and cellular metagenomes used for functional profiling of viral and cellular metagenomes using the KEGG Orthology database. Metagenomes obtained from the MG-RAST database were first analyzed by Dinsdale et al. (2009). (DOCX) [file pone.0109696.s013.docx]

**Table S6**. List of viral and cellular metagenomes used for functional profiling of viral and cellular metagenomes using the KEGG Orthology database. Metagenomes obtained from the MG-RAST database were first analyzed by Dinsdale *et al.* (2009).

| **Metagenome name** | **Accession number** | **Viral or Cellular** | **Type of biome** |
| --- | --- | --- | --- |
| Fish slime | 4440059.3 | Cellular | Fish |
| Fish slime | 4440065.3 | Viral | Fish |
| Healthy fish pond | 4440413.3 | Cellular | Freshwater |
| Healthy fish pond | 4440412.3 | Viral | Freshwater |
| High salinity salterns, west California | 4440419.3 | Cellular | Salt water |
| High salinity salterns, west California | 4440145.4 | Viral | Salt water |
| High salinity salterns, west California | 4440144.4 | Viral | Salt water |
| High salinity salterns, west California | 4440421.3 | Viral | Salt water |
| Highborne Cay | 4440061.3 | Cellular | Salt water |
| Highborne Cay | 4440323.3 | Viral | Salt water |
| Line Islands, Christmas Island | 4440041.3 | Cellular | Seawater |
| Line Islands, Christmas Island | 4440038.3 | Viral | Seawater |
| Line Islands, Kingman Island | 4440037.3 | Cellular | Seawater |
| Line Islands, Kingman Island | 4440036.3 | Viral | Seawater |
| Line Islands, Palmyra Island | 4440039.3 | Cellular | Seawater |
| Line Islands, Palmyra Island | 4440040.3 | Viral | Seawater |
| Line Islands, Tabuaeran | 4440279.3 | Cellular | Seawater |
| Line Islands, Tabuaeran | 4440280.3 | Viral | Seawater |
| Low salinity salterns, San Diego | 4440437.3 | Cellular | Salt water |
| Low salinity salterns, San Diego | 4440436.3 | Viral | Salt water |
| Low salinity salterns, San Diego | 4440432.3 | Viral | Salt water |
| Low salinity salterns, west California | 4440426.3 | Cellular | Salt water |
| Low salinity salterns, west California | 4440420.3 | Viral | Salt water |
| Medium salinity salterns, San Diego | 4440434.3 | Cellular | Salt water |
| Medium salinity salterns, San Diego | 4440435.3 | Cellular | Salt water |
| Medium salinity salterns, west California | 4440416.3 | Cellular | Salt water |
| Medium salinity salterns, west California | 4440425.3 | Cellular | Salt water |
| Medium salinity salterns, west California | 4440428.3 | Viral | Salt water |
| Medium salinity salterns, west California | 4440431.3 | Viral | Salt water |
| Medium salinity salterns, west California | 4440417.3 | Viral | Salt water |
| Medium salinity salterns, west California | 4440427.3 | Viral | Salt water |
| *Porites compressa* coral | 4440378.3 | Cellular | Coral |
| *Porites compressa* coral | 4440374.3 | Viral | Coral |
| Pozas Azules | 4440067.3 | Cellular | Microbialite |
| Pozas Azules | 4440320.3 | Viral | Microbialite |
| Rio Mesquites | 4440060.4 | Cellular | Microbialite |
| Rio Mesquites | 4440321.3 | Viral | Microbialite |
| Salton Sea | 4440329.3 | Cellular | Sediments |
| Salton Sea | 4440327.3 | Viral | Sediments |
| Salton Sea | 4440328.3 | Viral | Sediments |
| Tilapia Pond | 4440422.3 | Cellular | Freshwater |
| Tilapia Pond | 4440440.3 | Cellular | Freshwater |
| Tilapia Pond | 4440424.3 | Viral | Freshwater |
| Tilapia Pond | 4440439.3 | Viral | Freshwater |
