# Supplementary material for: Evolutionary Strategies of Viruses, Bacteria and Archaea in Hydrothermal Vent Ecosystems Revealed through Metagenomics
Source: PLoS One. 2014 Oct 3;9(10):e109696. doi: 10.1371/journal.pone.0109696 (PMC4184897; doi:10.1371/journal.pone.0109696)
Supplement: File S1 — Supplementary methods regarding the analysis of CRISPRs in the metagenomes. (DOCX) [file pone.0109696.s014.docx]

**SUPPLEMENTARY FILE 1**

***CRISPR analyses***

We identified Clustered Regularly Interspaced Palindromic Repeats (CRISPRs) [1–10] in assembled cellular metagenome reads to evaluate the degree to which these viruses actively infect cells in this study site. MetaCRT [11] identified 17 CRISPR loci containing 81 spacers from contigs assembled from the cellular metagenome. None of these spacers had matches to previously sequenced spacers in either our previously published CRISPR spacer database [12], or in the CRISPRdb database constructed by Grissa *et al.* [13]. We define a “match” as a 100% identity over at least 20 base pairs [12]. However, five of the spacers identified in the hydrothermal vent cellular metagenome contigs had matches to reads in the hydrothermal vent virome. All of the viral reads matching these CRISPRs were annotated as “unknown,” with the exception of one read labeled as a lipoprotein from *Fibrobacter succinogenes*. The matches between the cellular CRISPR spacers and the viral fraction indicate that the CRISPR loci of cells from the vent environment have recorded fairly recent infections by these viruses. Interestingly, the spacers that matched the vent virome occurred several times within the same CRISPR locus, and spacers located at the 5’ end of the CRISPR locus matched several different viral reads. The 5’ location of CRISPRs matching multiple viral reads supports previous findings that CRISPR spacers incorporate sequentially at the 3’ end of the locus, with the most conserved (and most frequently used) spacers occurring at the 5’ end [14].

1. Barrangou R, Fremaux C, Deveau H, Richards M, Boyaval P, et al. (2007) CRISPR provides acquired resistance against viruses in prokaryotes. Science (80- ) 315: 1709–1712. doi:10.1126/science.1138140.

2. Brouns SJ. J, Jore MM, Lundgren M, Westra ER, Slijkhuis RJ. H, et al. (2008) Small CRISPR RNAs guide antiviral defense in prokaryotes. Science (80- ) 321: 960 –964. doi:10.1126/science.1159689.

3. Sorek R, Kunin V, Hugenholtz P (2008) CRISPR — a widespread system that provides acquired resistance against phages in bacteria and archaea. Nat Rev Microbiol 6: 181–186. doi:10.1038/nrmicro1793.

4. Van der Oost, J., Jore, M.M., Westra, E.R., Lundgren, M., Brouns, S.J.J., et al. (2009) CRISPR-based adaptive and heritable immunity in prokaryotes. Trends Biochem Sci 34: 401–407. doi:10.1016/j.tibs.2009.05.002.

5. Horvath P, Barrangou R, Hovarth P (2010) CRISPR/Cas, the immune system of bacteria and archaea. Science (80- ) 327: 167–170. doi:10.1126/science.1179555.

6. Labrie SJ, Samson JE, Moineau S (2010) Bacteriophage resistance mechanisms. Nat Rev Microbiol 8: 317–327. doi:10.1038/nrmicro2315.

7. Marraffini LA, Sontheimer EJ (2010) Self versus non-self discrimination during CRISPR RNA-directed immunity. Nature 463: 568–571. doi:10.1038/nature08703.

8. Hale CR, Zhao P, Olson S, Duff MO, Graveley BR, et al. (2009) RNA-guided RNA cleavage by a CRISPR RNA-Cas protein complex. Cell 139: 945–956.

9. Jore MM, Lundgren M, van Duijn E, Bultema JB, Westra ER, et al. (2011) Structural basis for CRISPR RNA-guided DNA recognition by Cascade. Nat Struct Mol Biol 18: 529–536. doi:10.1038/nsmb.2019.

10. Garneau JE, Dupuis MÈ, Villion M, Romero DA, Barrangou R, et al. (2010) The CRISPR/Cas bacterial immune system cleaves bacteriophage and plasmid DNA. Nature 468: 67–71.

11. Rho M, Wu Y-W, Tang H, Doak TG, Ye Y (2012) Diverse CRISPRs evolving in human microbiomes. PLoS Genet 8: e1002441. doi:10.1371/journal.pgen.1002441.

12. Anderson RE, Brazelton WJ, Baross JA (2011) Using CRISPRs as a metagenomic tool to identify microbial hosts of a diffuse flow hydrothermal vent viral assemblage. FEMS Microbiol Ecol 77: 120–133.

13. Grissa I, Vergnaud G, Pourcel C (2007) The CRISPRdb database and tools to display CRISPRs and to generate dictionaries of spacers and repeats. BMC Bioinformatics 8: 172. doi:10.1186/1471-2105-8-172.

14. Andersson AF, Banfield JF (2008) Virus population dynamics and acquired virus resistance in natural microbial communities. Science (80- ) 320: 1047–1050. doi:10.1126/science.1157358.
